# Ectodysplasin/NF-κB Promotes Mammary Cell Fate via Wnt/β-catenin Pathway

**DOI:** 10.1371/journal.pgen.1005676

**Published:** 2015-11-18

**Authors:** Maria Voutilainen, Päivi H. Lindfors, Ewelina Trela, Darielle Lönnblad, Vera Shirokova, Teresa Elo, Elisa Rysti, Ruth Schmidt-Ullrich, Pascal Schneider, Marja L. Mikkola

**Affiliations:** 1 Developmental Biology Program, Institute of Biotechnology, University of Helsinki, Helsinki, Finland; 2 Max-Delbrück-Center for Molecular Medicine, Berlin, Germany; 3 Department of Biochemistry, University of Lausanne, Epalinges, Switzerland; University of Oxford, UNITED KINGDOM

## Abstract

Mammary gland development commences during embryogenesis with the establishment of a species typical number of mammary primordia on each flank of the embryo. It is thought that mammary cell fate can only be induced along the mammary line, a narrow region of the ventro-lateral skin running from the axilla to the groin. Ectodysplasin (Eda) is a tumor necrosis factor family ligand that regulates morphogenesis of several ectodermal appendages. We have previously shown that transgenic overexpression of Eda (*K14-Eda* mice) induces formation of supernumerary mammary placodes along the mammary line. Here, we investigate in more detail the role of Eda and its downstream mediator transcription factor NF-κB in mammary cell fate specification. We report that *K14-Eda* mice harbor accessory mammary glands also in the neck region indicating wider epidermal cell plasticity that previously appreciated. We show that even though NF-κB is not required for formation of endogenous mammary placodes, it is indispensable for the ability of Eda to induce supernumerary placodes. A genome-wide profiling of Eda-induced genes in mammary buds identified several Wnt pathway components as potential transcriptional targets of Eda. Using an ex vivo culture system, we show that suppression of canonical Wnt signalling leads to a dose-dependent inhibition of supernumerary placodes in *K14-Eda* tissue explants.

## Author Summary

Mammary glands are the most characteristic feature of all mammals. The successful growth and function of the mammary glands is vital for the survival of offspring since the secreted milk is the main nutritional source of a new-born. Ectodysplasin (Eda) is a signaling molecule that regulates the formation of skin appendages such as hair, teeth, feathers, scales, and several glands in all vertebrates studied so far. In humans, mutations in the *EDA* gene cause a congenital disorder characterized by sparse hair, missing teeth, and defects in exocrine glands including the breast. We have previously shown that excess Eda induces formation of supernumerary mammary glands in mice. Here, we show that Eda leads to extra mammary gland formation also in the neck, a region previously not thought to harbor capacity to support mammary development. Using Eda loss- and gain-of-function mouse models and transcriptional profiling we identify the downstream mediators of Eda. The presence of extra nipples is a fairly common developmental abnormality in humans. We suggest that misregulation of Eda or its effectors might account for some of these malformations. Further, the number and location of the mammary glands vary widely between different species. Tinkering with the Eda pathway activity could provide an evolutionary means to modulate the number of mammary glands.

## Introduction

The murine mammary gland development initiates at around embryonic day 10.5 (E10.5) with the establishment of bilateral milk or mammary lines [1]. Between E11-E12, five pairs of mammary placodes, local thickenings of the epithelium, emerge at conserved positions. By E13.5, the placodes have transformed via hillock stage to buds that have submerged downward and are surrounded by several layers of a specialized dermis, the primary mammary mesenchyme [1]. As the tip of the primordium begins to elongate, at E15.5, it forms a primary sprout that invaginates into the more distal secondary mammary mesenchyme. Branching morphogenesis begins a day later, and by birth a small ductal tree with several branches has formed.

The murine mammary line is not externally visible but only detectable from histological sections or molecularly identifiable by expression of Wnt pathway genes such as *Wnt10b* or TOP-gal, a transgenic reporter of the canonical Wnt pathway [2, 3]. Initially, the milk line is not a continuous structure but instead three independent *Wnt10b*-positive stripes arise: in axillary and inguinal regions, and the third one in the flank between the fore and hind limb buds. The axillary milk line gives rise to placode 1, inguinal to placode 5, and placodes 2, 3 and 4 form from the milk line of the flank [2]. Establishment of placodes is asynchronous and expression analysis of the Wnt pathway mediator *Lef1* revealed a designated order: 3, 4, 1/5 and 2 [4]. As the placodes form, low level of *Wnt10b* expression transiently combines all three milk lines but by E12.5 *Wnt10b* expression becomes confined to mammary buds [2, 3]. Placode morphogenesis is thought to rely mainly on migration of the progenitor cells along and from the immediate vicinity of the milk line and not on proliferation [5, 6].

Similar to other ectodermal appendages such as hair follicles and teeth, reciprocal interactions within and between the epithelium and the underlying mesenchyme are a necessity for proper development and pattering of mammary glands [1, 7, 8]. These interactions are mediated by conserved signaling pathways, of which at least the fibroblast growth factor (Fgf), Wnt/β-catenin, and Neuregulin (Nrg)/ErbB pathways regulate mammary placode formation. Mammary gland initiation relies on a complex interplay between these pathways and transcription factors Gli3 and Tbx3 and their absence disrupts formation of one or more placode pairs (reviewed in [9]).

Fgf10, emanating from the tip of the thoracic somites and the limb buds, has been proposed to function as one of the earliest signals for milk line specification. In the absence of *Fgf10* or its receptor *FgfR2b*, only placode pair four develops [2, 4]. Wnt/β-catenin signaling is required for all mammary placodes to form. Ectopic ectodermal expression of the secreted Wnt inhibitor Dkk1 abolishes all signs of mammary placodes [3]. Disruption of the Hedgehog pathway mediator Gli3 leads to loss of placodes 3 and 5 [10, 11]. In *Tbx3* null embryos, all mammary placodes are absent with the exception of occasional presence of placode 2 [12]. Tbx3 has been proposed to act both up- and downstream of Fgf and Wnt pathways but the details of these interactions are not well understood [12–14]. Finally, hypomorphic *Nrg3* mutant mice display frequently missing or hypoplastic placode 3 but also supernumerary placodes [15], whereas ectodermal overexpression of Nrg3 induces multiple supernumerary mammary glands along and adjacent to the milk line [16].

Another important player in embryonic mammary gland development is the tumor necrosis factor (Tnf) superfamily ligand Ectodysplasin-A1 (hereafter Eda) and its receptor Edar. The Eda pathway has a well characterized role in the development of diverse set of ectodermal organs [17, 18]. The ectodermal appendage phenotype of *Eda* null mice (*Tabby* mice) and mice with compromised activation of transcription factor NF-κB is highly similar [19], and biochemical and genetic studies have confirmed the importance of NF-κB downstream of Eda [18, 20]. In humans, mutations in the genes encoding EDA, EDAR, or the cytosolic signal mediator EDARADD cause a condition known as hypohidrotic ectodermal dysplasia (HED). In addition to tooth, hair, and sweat and salivary gland defects, breast anomalies such as hypoplastic/absent/supernumerary nipples and even absence of breast tissue have been reported in HED patients [21–23]. Studies using *Eda* loss- and gain-of-function mouse models have shown that Eda regulates embryonic and prepubertal mammary gland branching morphogenesis via NF-κB [24]. However, all five mammary glands form in *Eda* null mice suggesting that Eda is dispensable for mammary placode formation [24, 25]. Strikingly, ectodermal overexpression of Eda (*K14-Eda* mice) leads to formation of supernumerary mammary placodes along the milk line, in particular in the region between mammary buds 3 and 4, and give rise to supernumerary mammary glands in the adult [26, 27]. Beyond this, little is known about the importance of Eda in the initial stages of mammary gland development.

We report here that NF-κB is dispensable for mammary placode induction, yet it is necessary for the ability of Eda to induce supernumerary mammary primordia. Using an unbiased genome-wide approach, we identify several transcriptional targets of Eda. We provide evidence indicating that Eda promotes mammary cell fate by enhancing canonical Wnt signaling activity. Furthermore, we find that Eda induces supernumerary mammary glands not only between the endogenous mammary glands, but also in the neck region. Based on analysis of wild-type and *K14-Eda* embryos we propose that the murine mammary line extends more anteriorly than previously recognized.

## Results

### Supernumerary mammary buds form anterior to the mammary line in *K14-Eda* embryos

**Fig 1:**
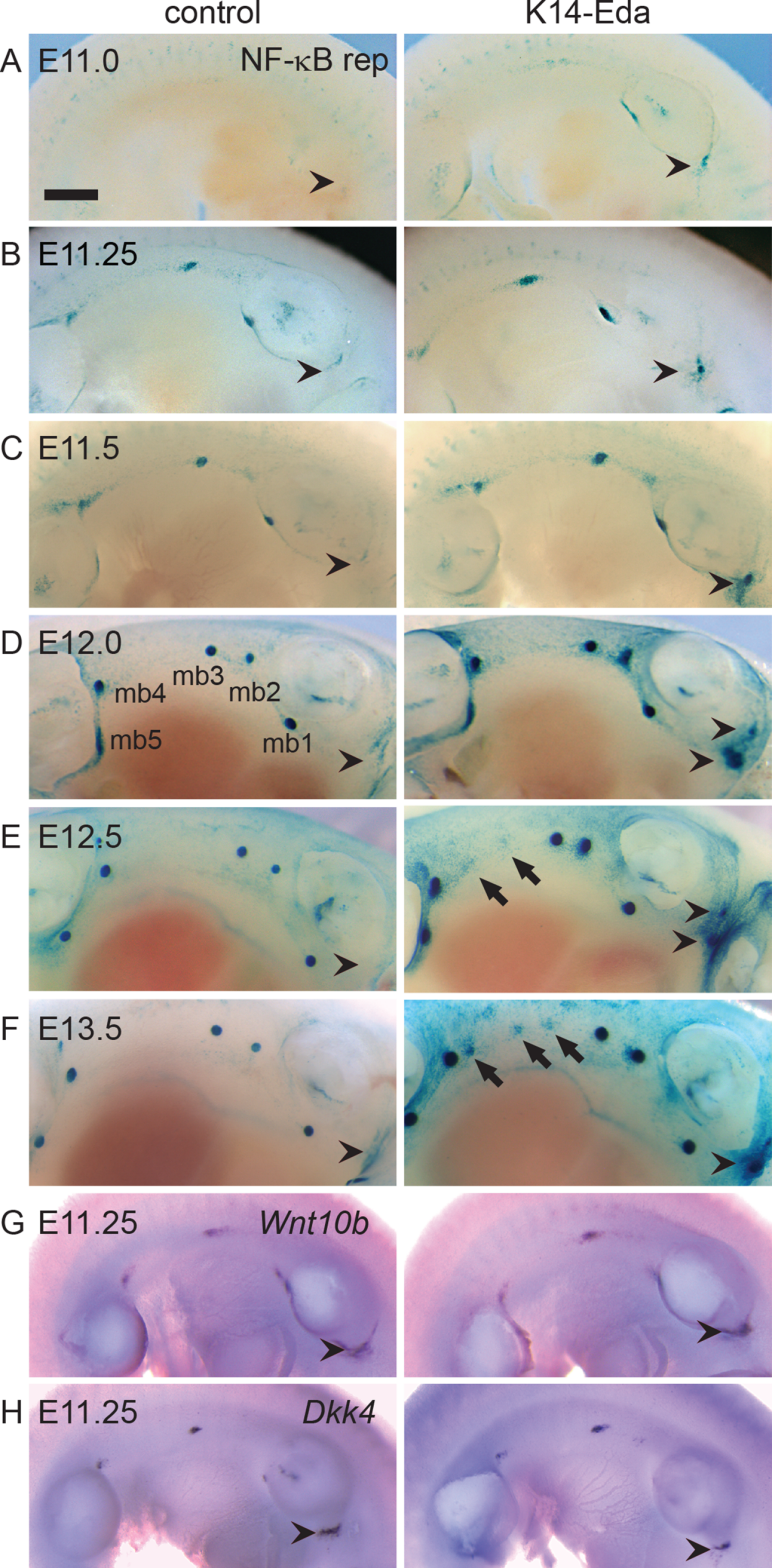
Overexpression of Eda induces formation of supernumerary mammary placodes in the neck and the flank. (A-F) NF-κB reporter expression was analyzed by X-gal whole mount staining in control and *K14-Eda* littermates at E11.0 (A), 11.25 (B), E11.5 (C), E12.0 (D), E12.5 (E), and E13.5 (F). In control, a low level NF-κB activity was observed anterior to the fore limb bud whereas the same region in *K14-Eda* gave rise to supernumerary X-gal positive foci (arrowheads). By E12.5 NF-κB became downregulated in the inter-placodal region in the control embryos whereas in *K14-Eda* embryos the appearance of supernumerary placodes between buds 3 and 4 started to become apparent at E12.5 and was more pronounced at E13.5 (arrows). (G-H) Whole mount in situ hybridization using digoxigenin-labelled probes specific to *Wnt10b* (G) and *Dkk4* (H) at E11.25. Expression was detected in the emerging mammary placodes, as well as anterior to the fore limb bud (arrowheads) in both genotypes. (Scale bar: 500 μm).

Embryonic mammary primordia exhibit high Eda-dependent NF-κB activity from E12 onwards [24, 25]. To gain further insights on the role of the Eda/NF-κB pathway in early mammogenesis, we assessed NF-κB signaling activity with reporter mice expressing β*-*galactosidase under an NF-κB–responsive element in control and *K14-Eda* embryos. We detected NF-κB activity in the mammary placode forming region from E11 onwards (Figs 1 and S1). At E11.0, a low level reporter expression was detected in the region of future mammary placode 3 and the interface of the forelimb bud and the thorax where placode 1 will later appear (Figs 1A and S1). At E11.25 faint expression was detected also at the border of the hind limb bud and ventrum (prospective placode 5), as well as at the site of future primordium 4 (Figs 1B and S1B). By E11.5 reporter expression had intensified in placode 3 and become more condensed at placodes 1, 4, and 5 (Fig 1C). Dispersed X-gal-positive cells were detected at the location of prospective placode 2 (Figs 1C and S1D). In addition, modest amount of reporter positive cells were observed along the entire milk line, from placode 1 to 5. At E12.0 high localized reporter expression was confined to the mammary buds and low level NF-κB activity was found throughout the dorsal side of the embryo whereas the ventrum appeared devoid of reporter expression (Fig 1D). At these early stages, the reporter expression was constantly stronger in *K14-Eda* background (Fig 1A–1D).

Similar to previous reports on expression of the Wnt pathway genes and TOP-gal reporter, NF-κB reporter positive cells disappeared from the milk line between E12.5 and E13.5 in control embryos (Fig 1E and 1F). In *K14-Eda* embryos, elevated NF-κB signaling was observed along the milk line, as well as in the dorsum, yet they exhibited no obvious focal clustering of X-gal-positive cells between buds 3 and 4, i.e. at the site of prospective supernumerary primordia, until at ~E12.5 (Fig 1C–1E). These foci were more pronounced at E13.5, although reporter expression was markedly less intense than in endogenous buds (Fig 1F).

The milk line, the area possessing mammary inductive capacity, is considered to extend from the axilla to the groin [1]. To our surprise, we observed faint NF-κB reporter activity from E11.0 onwards also in the neck area, anterior to mammary bud 1, which was substantially more pronounced in *K14-Eda* embryos (arrowheads in Fig 1). In *K14-Eda* embryos, reporter expression was confined to one or up to four small foci suggesting that supernumerary placodes were induced in the neck region. *In situ* hybridization analysis revealed high focal expression of *Wnt10b*, as well as *Dkk4*, another placode marker [28] in endogenous placodes of E11.25 wild type and *K14-Eda* embryos, as well as in the neck region (Fig 1G and 1H). The latter coincided with the site of ectopic placodes marked by NF-κB reporter expression in *K14-Eda* embryos (compare Fig 1G and 1H to Fig 1A–1C).

**Fig 2:**
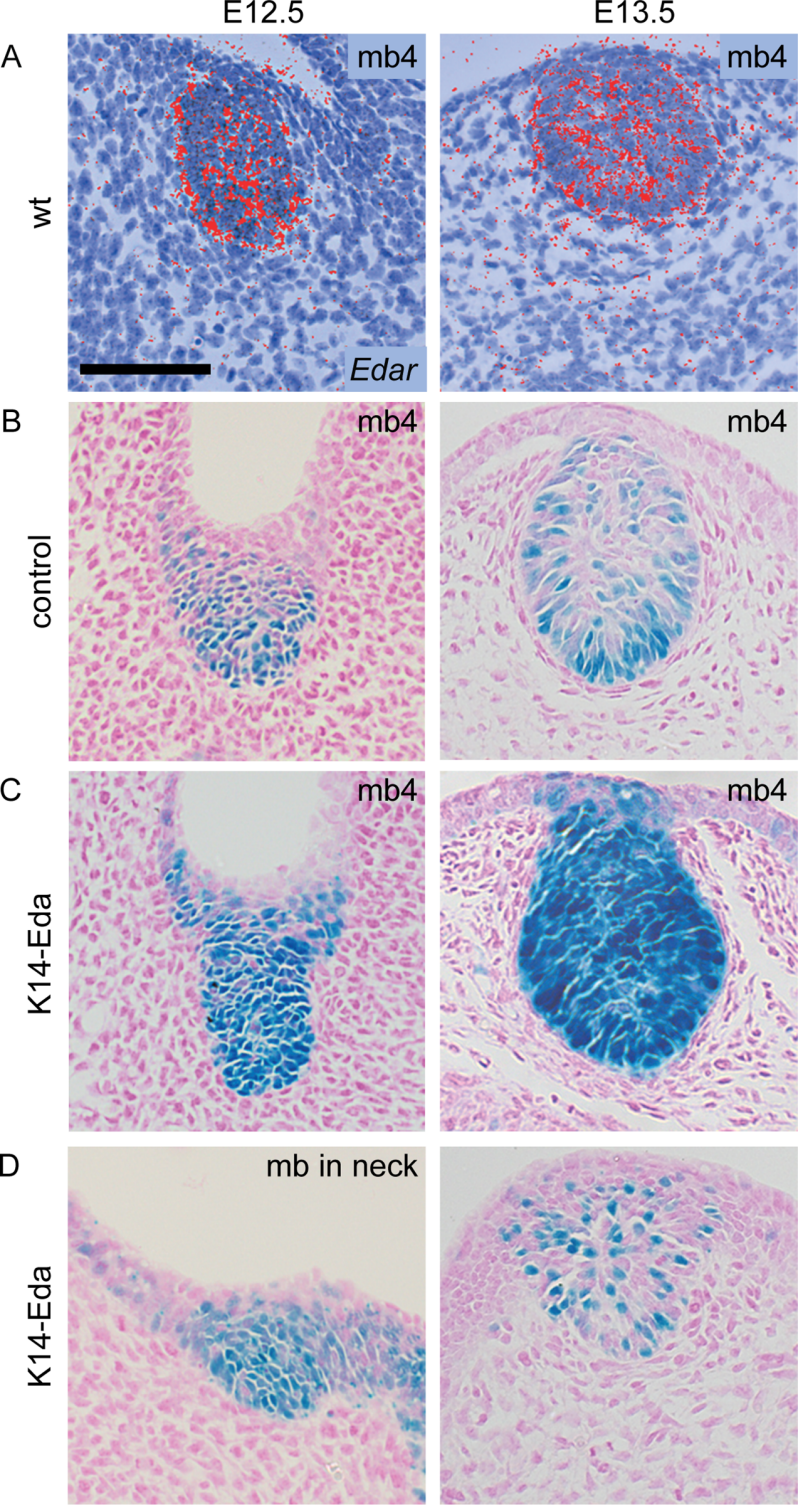
*Edar* expression and NF-κB reporter expression co-localize in the mammary epithelium. (A) *Edar* transcripts were detected by in situ hybridization with a ^35^S-UTP-labeled probe in mammary buds at E12.5 and E13.5. (B) NF-κB reporter was initially expressed throughout the mammary bud in control embryos, but became localized to the basal layer of the epithelium around E13.5. (C) In *K14-Eda* embryos, reporter activity stayed high throughout the mammary bud at E12.5 and E13.5. mb4 = mammary bud number 4. (D) The supernumerary buds exhibited mosaic expression of the reporter which was less pronounced in the in the neck (left) than in mammary primordia forming between buds 3 and 4 (right). (Scale bar: 100 μm.)

To analyze more in detail NF-κB activity, we sectioned whole mount stained reporter embryos (Fig 2). NF-κB activity was present throughout the developing mammary epithelium in control and *K14-Eda* embryos at E12.5, similar to expression of *Edar* (Fig 2A and 2B). At E13.5, NF-κB reporter activity was mainly confined to the basal cells in control embryos (Fig 2B), but remained high throughout the bud in *K14-Eda* embryos (Fig 2C). Further, sectioning confirmed that supernumerary neck placodes were truly thickened at E12.5 (Fig 2D, left column) and showed that supernumerary mammary buds, in particular those between buds 3 and 4, consisted of both reporter positive and negative cells (Fig 2D, right column).

### Supernumerary mammary buds in the neck give rise to ectopic mammary glands in the adult

**Fig 3:**
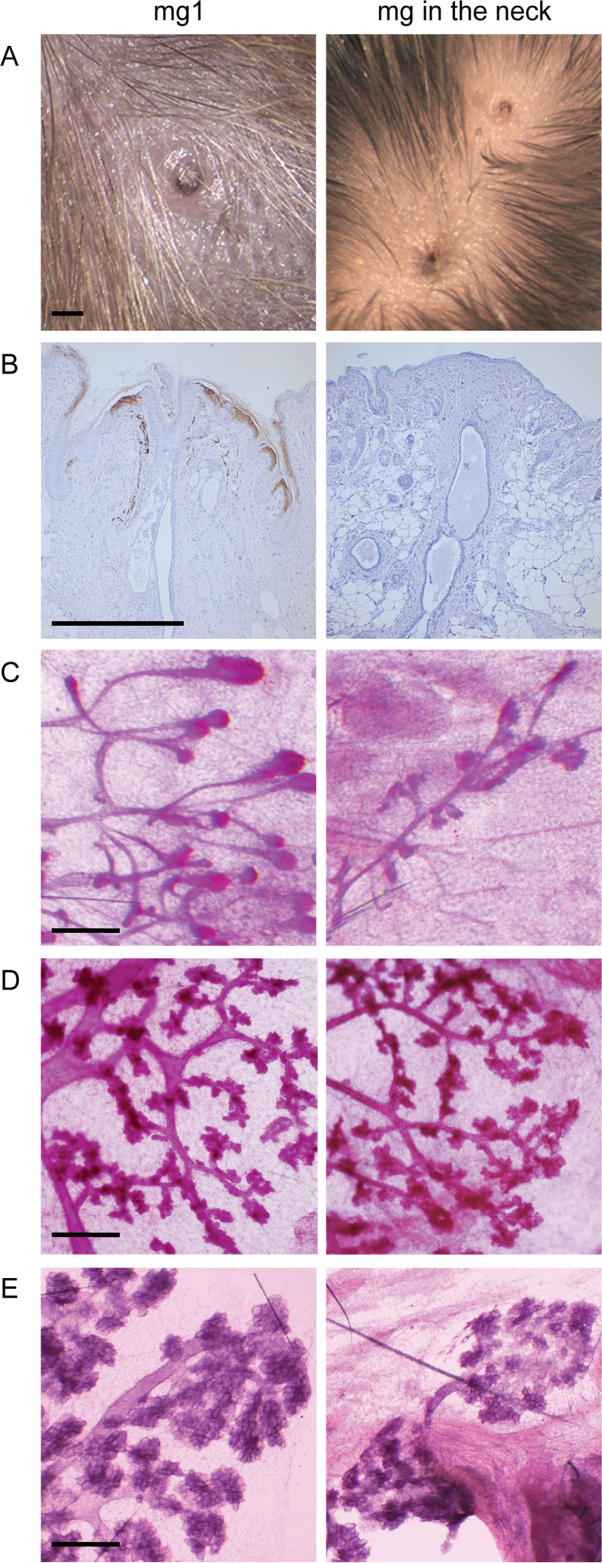
Supernumerary mammary gland in the neck consists of a nipple and a small ductal tree in *K14-Eda* mice. (A) Macroscopic view of the nipple of mammary gland 1 and two supernumerary nipples in the neck of a pregnant *K14-Eda* female. (B) Expression of the nipple marker keratin2e was readily observed in the endogenous but not in the supernumerary nipple. (C-E) Carmine alum stained ductal tree of mammary gland 1 and a supernumerary gland of K14-*Eda* female mouse at age of 5 weeks (C), on pregnancy day 14 (D), and day of parturition (E). (Scale bar: 500 μm)

Supernumerary mammary placodes forming between gland 3 and 4 give rise to nipples with an associated ductal system in *K14-Eda* adults, and are responsive to pregnancy hormones [26]. As suggested by embryonic analyses (Fig 1), a nipple was observed also in the neck region and was often accompanied by accessory, smaller nipple-like structures (Fig 3A). However, the nipple-like structures in the neck region did not express keratin 2e, a specific marker of nipple epithelium [29] indicating defective differentiation of the nipple epithelium (Fig 3B). Surprisingly, the neck region was also capable of supporting ductal morphogenesis (Fig 3C). Similar to the supernumerary glands located between glands 3 and 4 [26], the ductal trees in the neck were considerable smaller than those of the endogenous glands and displayed typical pregnancy-associated morphological changes (Fig 3D and 3E).

### NF-κB activity is dispensable for mammary placode formation

**Fig 4:**
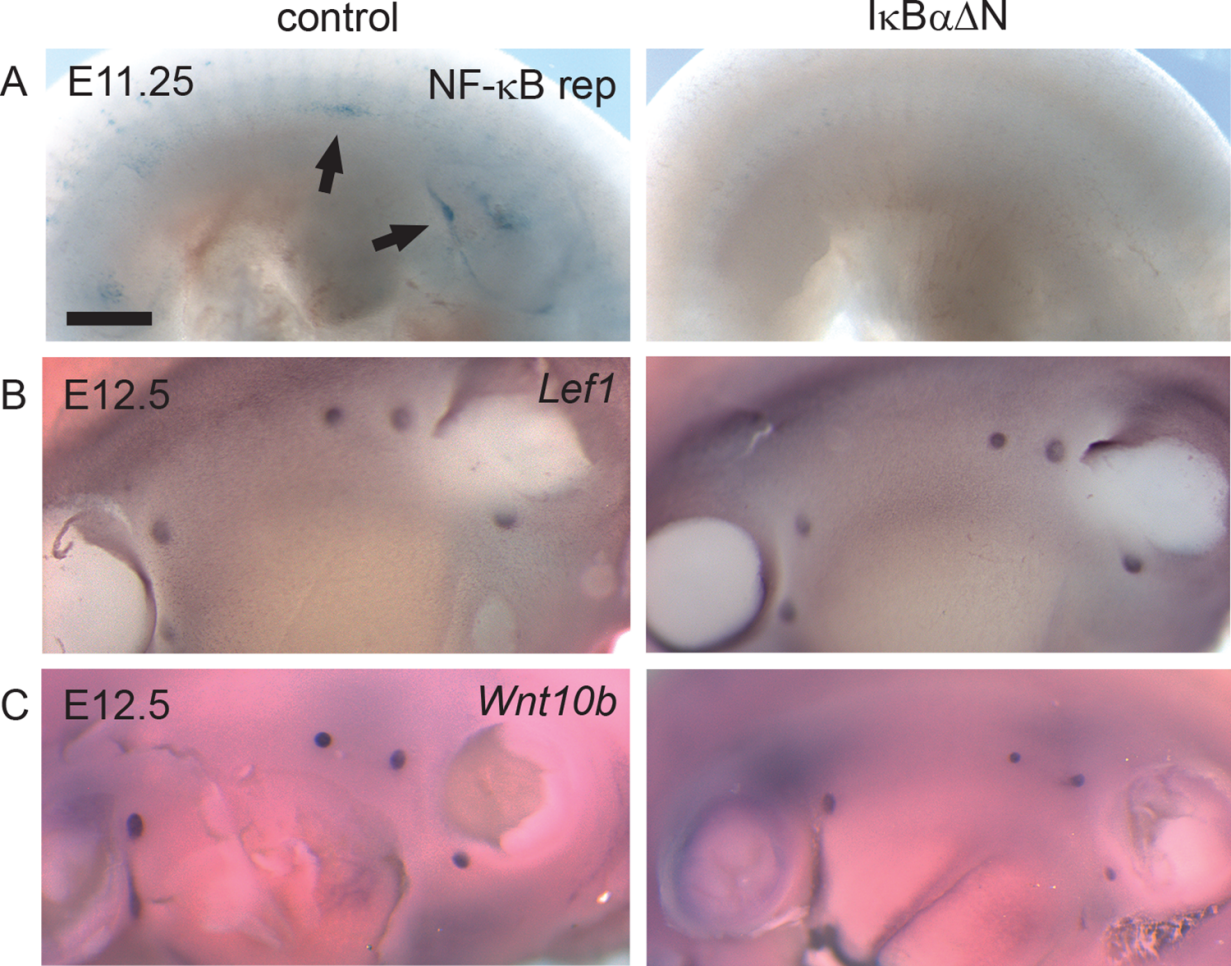
NF-κB is dispensable for mammary placode formation. (A) NF-κB reporter expression was analyzed in the emerging mammary placodes (arrows) of *IκBαΔN* and littermate control embryos at E11.25. Expression of (B) *Lef1* and (C) *Wnt10b* was detected in the mammary buds of both *IκBαΔN* and control embryos at E12.5 embryos. (Scale bar: 500 μm.)

As discussed above, engagement of Edar leads to activation of NF-κB. In unstimulated cells, inhibitory IκB proteins, most commonly IκBα, retain NF-κB is in the cytosol [30]. Ligand binding leads to phosphorylation and degradation of IκBα thereby releasing NF-κB. To elucidate the importance of NF-κB signaling in the embryonic mammary placode development, we utilized the *IκBαΔN* mouse strain which displays suppressed NF-κB activity as a result of ubiquitous expression of a non-degradable IκBα [19]. Analysis of NF-κB reporter expression in *IκBαΔN* embryos at E11.25 revealed absence of reporter expression (Fig 4A). This indicates that NF-κB signaling is fully suppressed in this mouse model at the time of mammary placode induction, similar to later developmental stages (E12-E16) [24]. In situ hybridization analysis of *Lef1*, which is expressed both in the mammary epithelium and the mesenchyme at E12.5 [31, 32], confirmed the presence of normal number of mammary primordia in *IκBαΔN* embryos (Fig 4B), yet *Wnt10b* expression suggested that mammary buds may be somewhat smaller (Fig 4C). Taken together, these data show that NF-κB activity is dispensable for mammary placode induction.

### Formation of Eda-induced supernumerary mammary placodes depends on NF-κB activity

**Fig 5:**
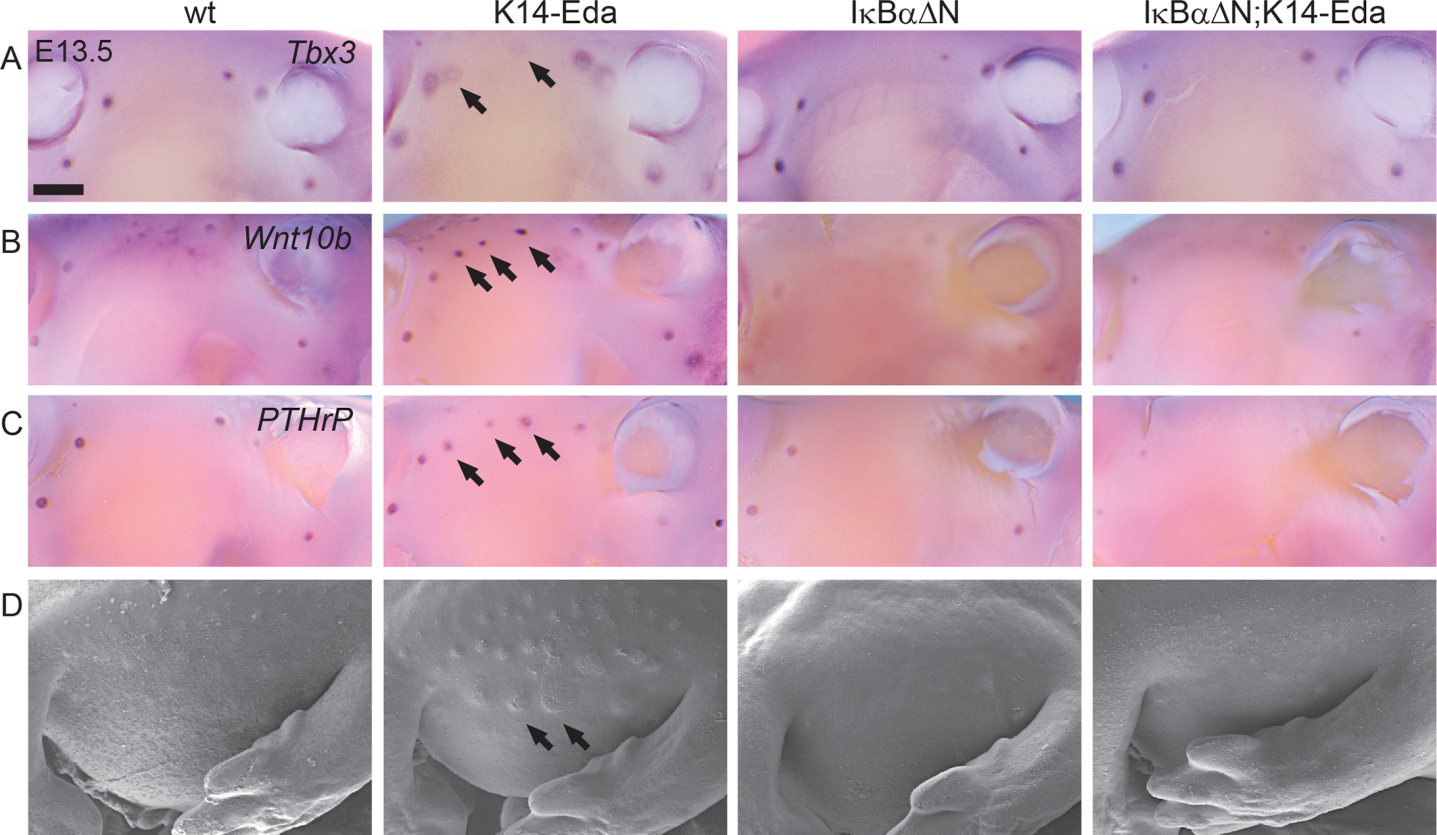
NF-κB is required for the formation of Eda induced supernumerary mammary placodes. (A-C) Expression of *Tbx3* (A), *Wnt10b* (B) and *PTHrP* (C) was detected in the mammary buds of the WT, *K14-Eda*, *IκBαΔN* and compound *IκBαΔN*; *K14-Eda* embryos at E13.5. Arrows highlight the supernumerary mammary placodes in *K14-Eda* embryos. Note that no localized expression was visible between the 3^rd^ and the 4^th^ mammary bud in the compound *IκBαΔN*; *K14-Eda* embryos. (D) Scanning electron microscopy images of the mammary forming region confirmed absence of accessory mammary primordia in compound mutants. (Scale bar: 500 μm.)

To address the necessity of NF-κB for the ability of Eda to induce supernumerary placodes, we crossed *K14-Eda* strain with the *IκBαΔN* mice. Mammary placode markers *Tbx3*, *Wnt10b*, and *PTHrP* [2, 24, 33] were expressed in mammary buds of wild type, *IκBαΔN*, *K14-Eda* and compound *K14-Eda*;*IκBαΔN* embryos at E13.5 (Fig 5A–5C). Expectedly expression of *PTHrP* and *Wnt10b* appeared slightly downregulated in *IκBαΔN* background as they have been identified to be transcriptional targets of Eda/NF-κB [24, 34]. All three were also detectable in the supernumerary primordia of *K14-Eda* embryos, *Tbx3* showing a circular expression pattern around the placodes though. Expression of all marker genes was completely abolished in the supernumerary placode forming region in the compound mutants (Fig 5A–5C). Analysis with scanning electron microscope (SEM) showed no morphological signs of supernumerary placodes in *K14-Eda;IκBαΔN* mutants (Fig 5D). Further, supernumerary nipples or ductal trees were never observed in the adult compound mutants. Our findings show that even though NF-κB is not needed for the formation of endogenous mammary placodes, it is indispensable for formation of Eda-induced supernumerary mammary placodes.

### A genome-wide screen identifies several putative transcriptional targets of Eda

In order to identify the immediate downstream targets of Eda/NF-κB, we performed microarray profiling of genes expressed in *Eda-/-* E13.5 mammary buds exposed to control medium or to recombinant Fc-Eda protein. Using the same setup, but quantitative real-time reverse-transcriptase–PCR (qRT-PCR) and candidate gene approach, we have previously shown that Eda upregulates expression of *Wnt10a*, *Wnt10b*, *Dkk4*, and *PTHrP* in mammary buds [24]. Altogether 245 probes were upregulated (including *Wnt10a*, *Wnt10b*, *Dkk4*, and *PTHrP*) and 78 probes downregulated by Eda treatment (Tables 1 and S1).

**Table 1:** List of selected upregulated genes from the microarray.

	Gene	log2
**Wnt pathway**	Dkk4	2,00
	Wnt10b	1,13
	Wnt10a	0,94
	Lrp4	0,93
	Kremen2	0,81
	Lef1	0,46
	Lgr4	0,35
**Fgf pathway**	Fgf17	0,97
	Dusp6	0,83
	Fgf20	0,35
**Chemokine pathway**	Cxcl10	1,63
	Cxcl2	1,48
	Cxcl11	1,29
	Cxcl9	1,07
	Cxcr4	0,73
**Other genes**	Clca2	2,3
	Clca1	1,8
	Madcam1	1,7
	Foxi3	1,43
	Adamts15	1,13
	Mmp9	1,06

Log2 indicates (log2(Fc-Eda-treatment)-log2(control)).

**Fig 6:**
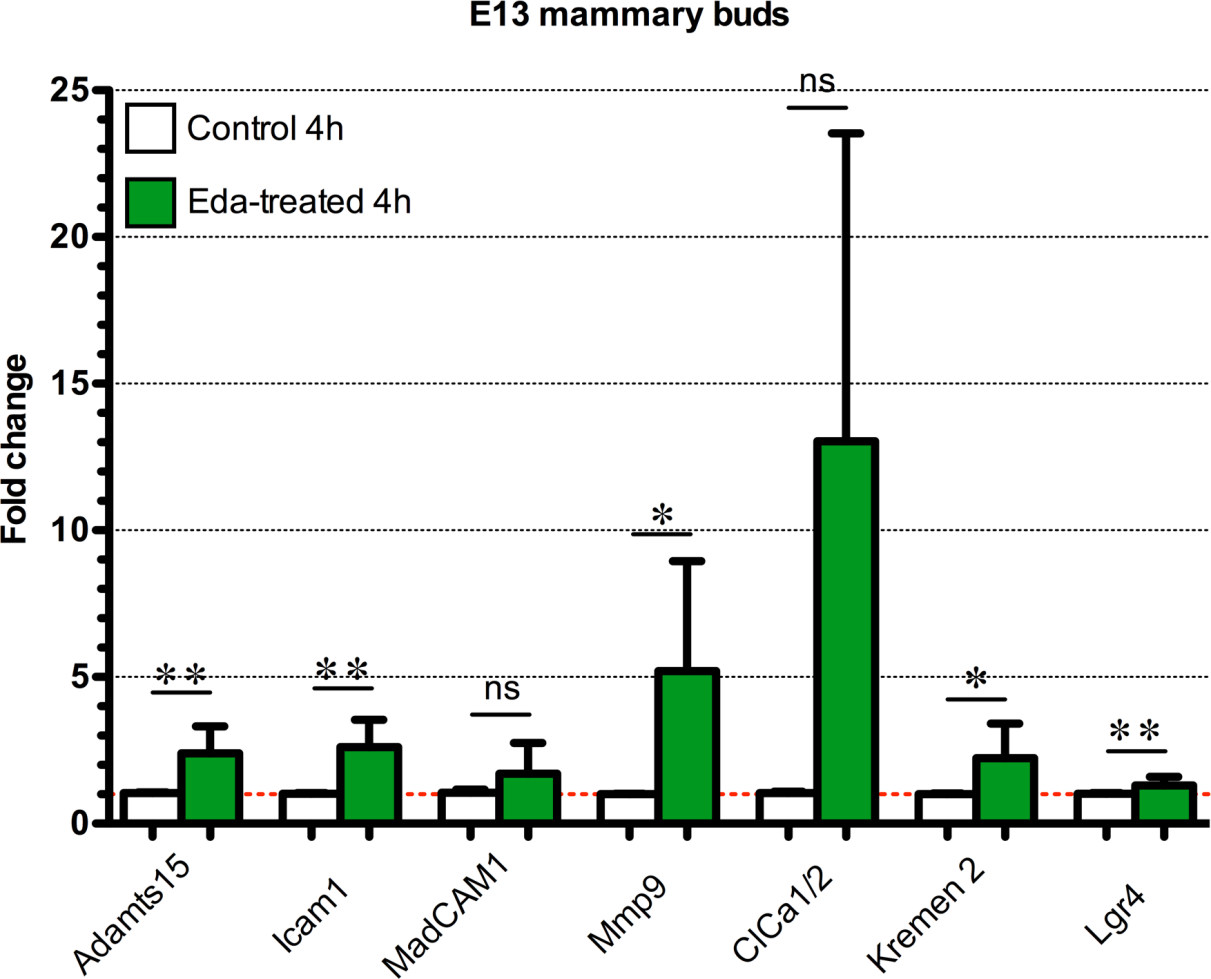
Quantitative RT-PCR analysis of putative Eda target genes in the mammary bud. E13.5 *Eda-/-* mammary primordia were treated with control medium or 250ng/mL of Fc-Eda for 4h and expression of the indicated genes was analyzed by qRT-PCR. Data are shown as mean ±SD. *P < 0.05; **P<0.01. *Adamts15*_(n = 6)_, *Icam1*_(n = 5)_, *Madcam1*_(n = 6)_, *Mmp9*_(n = 6)_, *ClCa1/2*_(n = 4)_, *Krm2*_(n = 6)_ and *Lgr4*_(n = 10)_.

Genes in several different signaling pathways including Wnt, Fgf, Tnf, Tgfβ, chemokine, and hedgehog pathways were differently expressed. In addition, adhesion molecules *Madcam1* and *Icam1*, extracellular matrix degrading metalloproteinases *Adamts15* and *Mmp9*, chloride channel proteins *clca1* and *clca2* (recently reannotated as a1 and a2 variants of clca3, respectively), and transcription factor *Foxi3* were among the upregulated genes (Table 1). To validate the microarray results, we performed qRT–PCR analysis and in situ hybridization (ISH) or immunostaining of selected genes, both strongly and modestly induced ones (Table 1, Figs 6A, 6B and S2). Of the 7 genes tested all showed the same tendency as in the microarray, the difference between control and Eda-treated specimen being statistically significant for 5 genes.

Both Madcam1 and Icam1 are known to be expressed in hair placodes and their transcripts are upregulated by Eda in E14 back skin [35]. We did not detect Madcam1protein or *Icam1*, *Adamts15*, or *Mmp9* transcripts in developing mammary primordia of control embryos by whole mount analysis, yet Madcam1 and *Mmp9* (but not *Icam1 or Adamts15*) were readily observed in *K14-Eda* embryos (S2A and S2B Fig) suggesting that they lie downstream of Eda in mammary buds. *Mmp9*-deficient mice have no overt mammary gland phenotype [36] possibly owing to redundancy with other Mmps. There are no reports on the function of the other genes in mammary gland development.

Transcription factor *Foxi3* was one of the most highly induced genes by Eda. *Foxi3* is mutated in several dog breeds, a condition described as canine ectodermal dysplasia [37]. We have previously identified *Foxi3* as an Eda-induced gene in developing hair follicles and teeth and shown augmented expression in *K14-Eda* mammary buds in vivo [38]. The finding prompted us to analyze whether Foxi3 could play a role in mammary gland induction. However, the mammary glands of *Foxi3* null embryos were indistinguishable from control littermates and formation of Eda-induced supernumerary mammary primordia was unaffected by loss of Foxi3 (S3A–S3D Fig).

### Wnt pathway genes are upregulated within the milk line prior to ectopic placode formation

Our microarray and previous qRT-PCR analyses revealed that several Wnt pathway genes are induced by Eda (Fig 5, [24]). Further, we have earlier reported that *Lef1* is expressed very early on in the emerging supernumerary placodes of *K14-Eda* embryos [27]. Given the importance of the Wnt pathway in mammary placode formation, we wanted to study more closely whether expression of the Wnt pathway genes is altered in response to diverse levels of Eda by comparing *Eda-/-*, wild type, and *K14-Eda* embryos at E12.5, when ectopic placodes are becoming apparent between buds 3 and 4.

**Fig 7:**
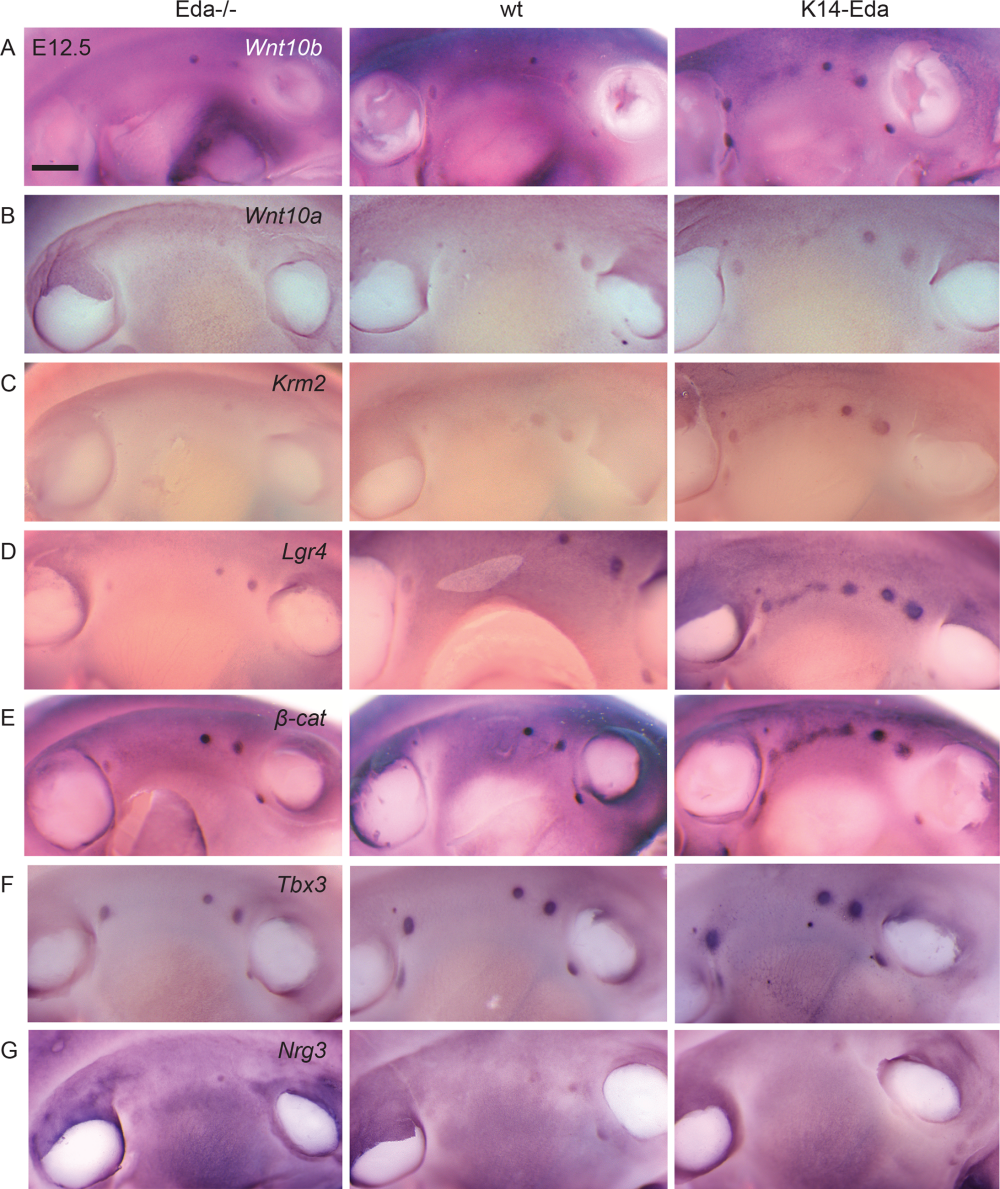
Wnt pathway genes are upregulated already at E12.5 in the region where supernumerary mammary placodes form. Expression of (A) *Wnt10b*, (B) *Wn10a*, (C) *Kremen2*, (D) *Lgr4*, (E) *β-cat*, (F) *Tbx3*, and (G) *Nrg3* in *Eda*-/-, wild-type and *K14-Eda* embryos. Note correlation of levels of *Wnt10b*, *Wnt10a*, *Kremen2*, and *Lgr4* with the Eda status (A-D).Further, *Wnt10b*, *Kremen2*, *Lgr4*, and *β-cat* show an early stripe-like expression pattern in the region where supernumerary mammary placodes emerge (A, C-E). (Scale bar: 500 μm.)

*Wnt10b*, one of the earliest markers of the milk line, becomes gradually restricted to the placodes as they emerge [2, 3]. Kremen2 (Krm2) is a transmembrane protein that inhibits Wnt signaling in the presence of Dkk proteins [39] whereas Lgr4 is a receptor for R-spondins, which are potent Wnt pathway stimulators [40, 41]. Both *Krm2* and *Lgr4* have been localized to E12.5 mammary buds [3, 42]. *Wnt10a*, *Wnt10b*, *Krm2* and *Lgr4* were all present in the endogenous mammary buds of all three genotypes (Fig 7A–7D). Expression of all four genes revealed a correlation with Eda levels: reduction in *Eda-/-* and up-regulation in *K14-Eda* mammary buds. Notably, *Wnt10b* and occasionally *Lgr4* and *Kremen2* were clearly upregulated as a continuous streak in *K14-Eda* embryos at the site where supernumerary placodes form. Further, we analyzed expression of *β-catenin*, which also exhibited a streak-like expression pattern between buds 3 and 4 in *K14-Eda* embryos (Fig 7E).

Next, we studied the expression of other genes critical for mammary placode formation. *Tbx3* and *Nrg3* are first detected in the mesenchyme but at the onset of placode formation they become upregulated (*Tbx3*) or completely restricted (*Nrg3*) to the mammary epithelium [12, 15, 33]. Expression of both genes was expectedly found in the endogenous mammary buds in all three genotypes (Fig 7F and 7G). However, neither of them could be detected in the ectopic mammary forming region in *K14-Eda* embryos at E12.5 (Fig 7F and 7G), yet *Tbx3* was observed in the ectopic primordia at E13.5 ([24]; Fig 5).

Our results show that all Wnt pathway genes studied exhibited early upregulation in the region where ectopic mammary placodes arise, whereas expression of other genes implicated in mammary placode formation (*Tbx3*, *Nrg3*) was detectable in this region only at a later developmental stage. Although different probes cannot be directly compared with each other, these data might suggest that especially Wnt pathway activation is critical for induction of ectopic placodes downstream of Eda. Further, our microarray associated Eda with several Wnt pathway genes, but revealed no link between Eda and *Tbx3* or *Nrg3*. Two Fgf ligands (*Fgf17* and *Fgf20*) were upregulated by Eda, but these Fgfs are thought to signal mainly via the mesenchymally expressed c isoforms of Fgfrs, not Fgfr2b [43], and are thus unlikely to function in a manner similar to Fgf10.

### Eda induces supernumerary placodes ex vivo

**Fig 8:**
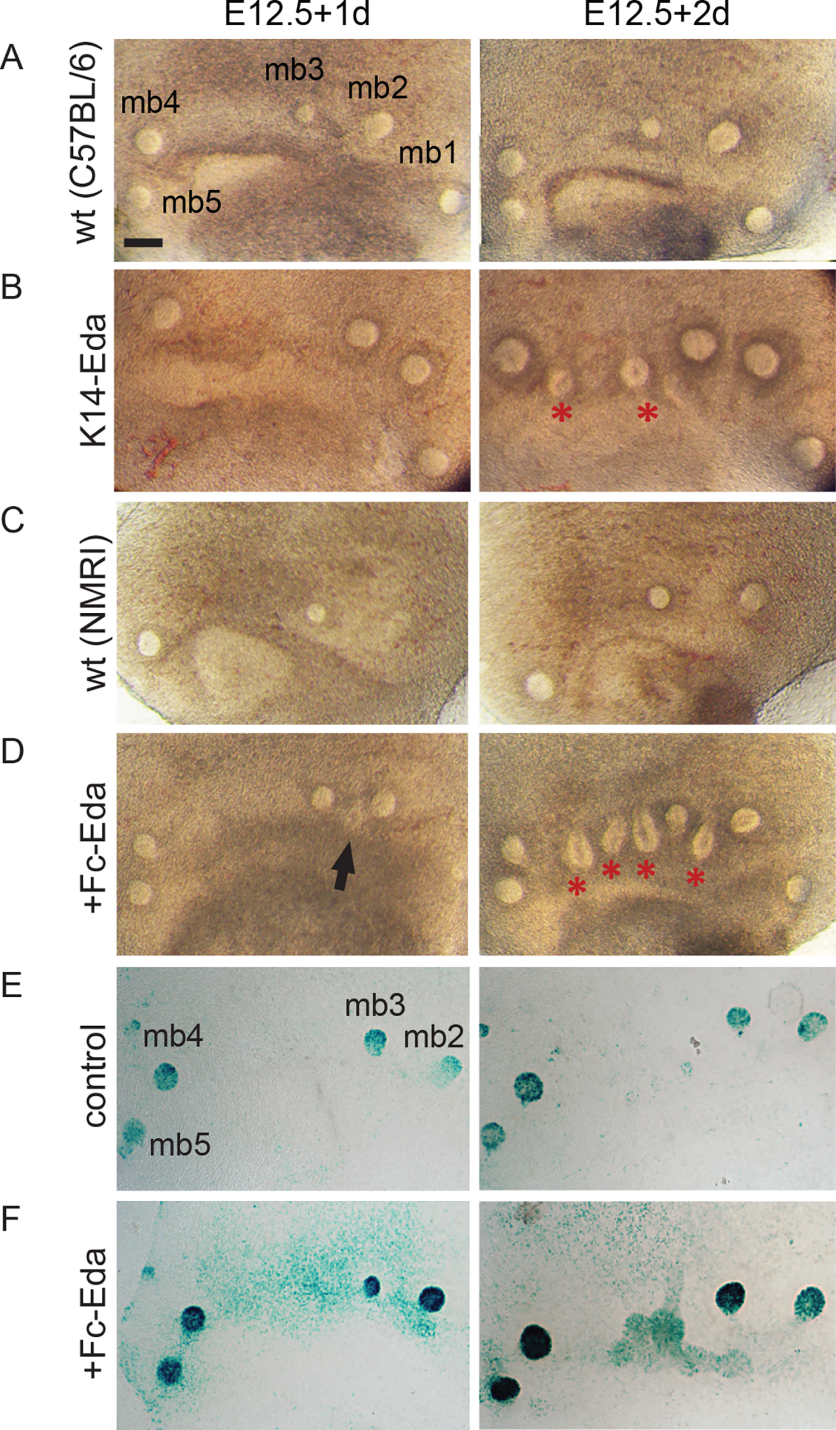
Recombinant Eda protein induces supernumerary mammary placodes and upregulates NF-κB reporter expression ex vivo. (A-D) Stereomicroscope images of E12.5 flank skins of WT_C57BL/6_ (n = 11) (A), *K14-Eda* (n = 15) (B), WT_NMRI_ (n = 21) (C) and WT_NMRI_ supplemented with 250 ng/mL of Fc-Eda (n = 42) (D) cultured for two days ex vivo. A and C are control littermates of B and D, respectively. Ectopic buds were observed in 0/32 control, 15/15 *K14-Eda*, and 38/42 Fc-Eda treated explants. (E-F) NF-κB reporter expression was analysed in E12.5+1d and E12.5+2d explants cultured in the control medium (E) and medium supplemented with 250ng/mL of Fc-Eda (F), by X-gal staining. (Scale bar: 100 μm.)

In order to be able to manipulate and follow mammary placode formation more precisely, we developed an ex vivo tissue culture setup. In brief, ventrolateral skin explants containing the milk line region were dissected from E12.5 embryos and grown in a Trowell-type culture system as described previously [44]. Explants isolated from *K14-Eda* and control littermate embryos were cultured for a period of two days. After one day (E12.5+1d), wild type and *K14-Eda* samples appeared almost identical (Fig 8A and 8B). By E12.5+2d, *K14-Eda* explants were clearly distinguishable from controls due to the presence of supernumerary bud-like structures that had formed between buds 3 and 4, and occasionally also between buds 2 and 3 (Fig 8A and 8B). Typically, 2–3 supernumerary primordia formed between buds 3 and 4. The explants thus recapitulated the in vivo phenotype very closely [27].

Next, we tested whether recombinant Fc-Eda protein had the capacity to induce formation of ectopic buds ex vivo. After one day (E12.5+1d), control and Eda-treated samples appeared fairly similar (Fig 8C and 8D) although incipient supernumerary placodes were observed in Eda-treated specimen. A day later, similar to *K14-Eda* explants, several ectopic bud-like structures had developed within the milk line in Eda-treated specimen, whereas the controls showed no morphological changes in this region (Fig 8C and 8D). Increased NF-κB reporter activity was evident in response to Eda treatment at the sites of presumptive supernumerary placodes (Fig 8E and 8F).

The endogenous mammary buds of both control and Eda-treated explants expressed *Wnt10b*, *Krm2* and *PTHrP*. As in vivo, expression of these genes was observed in Eda treated samples between buds 3 and 4 (S4A–S4C Fig). *Sonic hedgehog* (*Shh*) is a hair lacode-specific marker whose expression is barely detectably in mammary buds [45]. No *Shh* expression was observed in endogenous buds or in the region where supernumerary mammary primordia formed in control or Eda-treated samples (S4D Fig).

### Formation of supernumerary mammary placodes is dependent on Wnt activity

**Fig 9:**
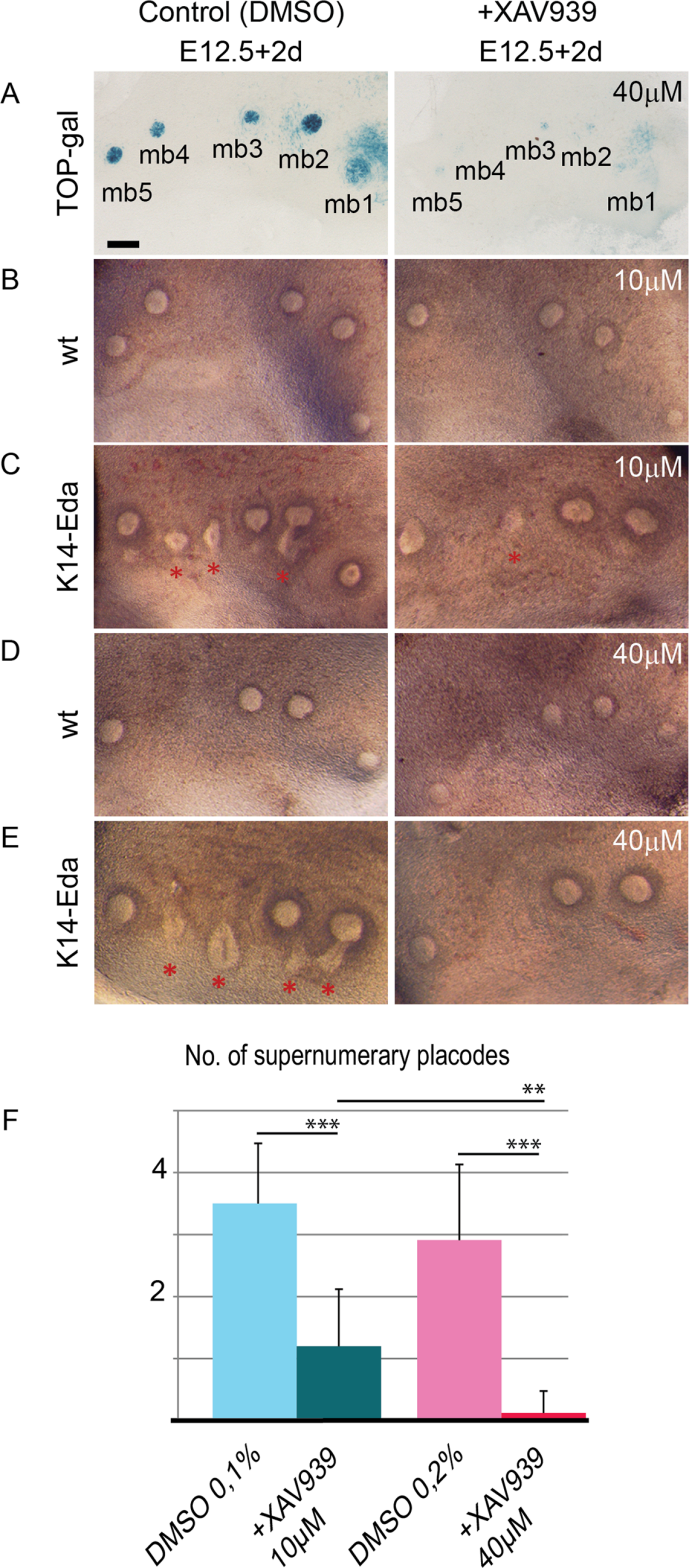
Inhibition of Wnt activity suppresses supernumerary mammary placode formation in a dose-dependent manner in *K14-Eda* tissue explants. (A-E) Stereomicroscope images of E12.5 TOP-gal (A) (n = 17), WT (B, D) and *K14-Eda* (C, E) explants cultured for 2 days in the presence of 10 μM XAV939 or equal amount of solvent (0.1% DMSO) (B, C), and 40 μM XAV939 or an equal amount of solvent (0.2% DMSO) (A, D, E). Supernumerary placodes were never observed in WT specimen (0/18 explants). (F) Quantification of supernumerary placodes in *K14-Eda* explants: data are shown as mean ±SD. ***P < 0.001, **P < 0.01. (B, C) WT _(n = 10)_ and *K14-Eda*_(n = 10)_; (D, E) WT _(n = 8)_ and *K14-Eda*_(n = 8)_. In all experiments, one side of the embryo was used as the control and the other was treated with XAV939. (Scale bar: 100 μm.)

Upregulation of several Wnt pathway genes by Eda suggests involvement of canonical Wnt signaling in the induction of supernumerary placodes. However, the effects of Wnt pathway would be difficult to assess genetically due to several putative target genes of Eda that could act redundantly. Instead, we cultured E12.5 wild type and *K14-Eda* explants in the presence of XAV939, an inhibitor of the canonical Wnt-pathway [46]. Application of XAV939 on tissues of TOP-gal Wnt reporter embryos confirmed significant downregulation of Wnt signaling in all treated explants (17/17 explants) (Fig 9A). Supernumerary placodes were always observed in non-treated *K14-Eda* samples at E12.5 + 2d whereas their formation was greatly reduced by low (10 μM) and almost completely inhibited by high (40 μM) concentration of XAV939, respectively (Fig 9B–9F). At these concentrations, XAV939 had no apparent effect on endogenous buds in wild-type or *K14-Eda* explants. We also tested the effect of XAV939 on endogenous placodes at the time when they emerge (E11.0) and visualized forming mammary primordia with the aid of K17-GFP transgene [47]. 40 μM of XAV939 did not prevent formation of endogenous placodes, although placode size was clearly reduced (S5 Fig) indicating that supernumerary placodes are more sensitive to Wnt inhibition than endogenous ones. In conclusion, these data suggest that Eda signaling upregulates Wnt activity within the milk line which leads to formation of ectopic mammary placodes.

## Discussion

### Murine milk line extends anterior to mammary gland 1

We report here that mice overexpressing the Tnf-like ligand Eda develop supernumerary mammary glands not only along the milk line [26, 27], but additionally in the neck region, anterior to mammary gland 1. Further, based on pregnancy-associated morphological changes, these glands are functional. Traditionally the region possessing mammary potential has been thought to be limited to the area between the axilla and the genital tubercle [1]. The murine mammary line has been identified by a streak of *Wnt10b* expressing cells [2]. Initially three separate streaks form: a central streak between the limbs appears first followed by independent stripes at the ventral border of each of the limbs where placodes 1 and 5 form [2]. We also observed similar stripes of NF-κB reporter expressing cells in the axilla and groin, and as reported for *Wnt10b* [2], they only later became connected with the central streak of the milk line.

In addition to the mammary line, a separate streak of *Wnt10b*-positive cells, named the dorsal line, has been noted but the importance of these cells has remained elusive [2]. This streak is located dorsally to the milk line, encircles the fore limb bud from the dorsal side and ends at the anterior edge of the fore limb bud. Intriguingly, this is exactly where ectopic placodes form in *K14-Eda* embryos. We observed a discernible cluster of *Wnt10b*-positive and *Dkk4*-positive cells, and a less pronounced aggregate of NF-κB reporter expressing cells, in this location also in control embryos. Inspection of published pictures reveals that this domain is also positive for several other placode markers including *Wnt6*, *Tbx3*, and s-SHIP-GFP [2, 33, 48]. Further, it is characterized by high TOP-Gal activity [3, 49]. Collectively, these data and our new findings indicate that the murine milk line extends past the axillary area.

Our data suggest that Eda induced mammary cell fate supporting signals result in the maintenance of a normally transient group of mammary cells at the far end of the dorsal line. Similarly, Eda has been proposed to sustain a transient signaling center in the dental lamina resulting in the formation of an ectopic tooth in *K14-Eda* mice [27]. On the other hand, in wild-type embryos the area between buds 3 and 4, another region where supernumerary mammary glands develop in *K14-Eda* mice, is characterized by scattered rather than clustered *Wnt10b*-positive cells. This may explain why Eda can readily overcome the developmental threshold for placode induction in the neck region leading to the appearance of supernumerary placodes in this position substantially earlier than elsewhere in the milk line. At the time when supernumerary placodes arise between buds 3 and 4, the streak of *Wnt10b*-positive cells in no longer detectable in control embryos, but it is maintained/reappears in *K14-Eda* embryos. However, similar to the endogenous buds, *Wnt10b* expression becomes later confined to the newly formed buds. This suggests that similar mechanisms account for the formation of both endogenous and supernumerary mammary primordia.

### NF-κB is needed for supernumerary mammary placode formation

Our analysis on *Eda-/-* embryos showed that lack of Eda does not interfere with the patterning of endogenous mammary placodes. NF-κB is thought to be activated by all Tnf receptors, but also JNK and p38 pathways can be employed by many Tnfrs [50]. JNK pathway has been suggested to mediate Edar signaling, at least in some cultured cell lines [51]. Though NF-κB can be activated by multiple stimuli we found no evidence that other NF-κB activating cues besides Eda operate during mammary placode formation. As NF-κB was shown to be dispensable for mammary gland induction, it was surprising that formation of Eda-induced supernumerary placodes was NF-κB -dependent. We find it plausible that Eda/NF-κB has a role in the formation of endogenous mammary placodes as well, but loss of function may be compensated for by other pathways, in particular those that enhance Wnt signaling activity (see also below). Nrg3/ErbB4 is one potential pathway that could exert this function as Nrg3-coated beads induce *Lef1* expression concomitant with ectopic placodes-like structures *in vitro* [15].

### Eda regulates genes in multiple pathways implicated in mammary gland development

Our microarray profiling of genes differentially expressed in mammary buds upon short exposure to recombinant Eda protein revealed members of several signaling pathways. This implies that Eda may act by tinkering the activity of multiple mammary-associated pathways during morphogenesis. We found significant changes in expression of Wnt, Fgf, Tnf, and chemokine pathway genes, similar to our previous findings in Eda-regulated genes in hair placodes [35, 52]. Although these two studies cannot be directly compared due to different microarray platforms used, it seems that the gene regulatory network governed by Eda is largely shared between hair follicles and mammary glands.

We found changes in several Wnt pathway genes upon Eda treatment; both agonists (*Wnt10a*, *Wnt10b*, *Lef1*, and *Lgr4*) and antagonists (*Lrp4*, *Kremen2* and *Dkk4*) were upregulated, as also observed in hair placodes [24, 34, 52]. Proper spacing of many ectodermal appendages is believed to be achieved by combinatory regulation of positive and negative signals [53, 54]. The reaction-diffusion model suggests that soluble factors that either promote or inhibit placode fate are co-expressed in placodes. However, unequal diffusion/stability of these substances may result in higher activator activity in the placodes whereas in the surrounding tissue, the opposite, higher inhibitor to activator ratio, prevents acquisition of placode fate. Further, it seems plausible that these cues are fine-tuned by several different pathways. The ability of Eda to modulate the expression of both placode activators and inhibitors in combination with the input from other signaling pathways may also explain the puzzling finding that HED patients may have both missing and supernumerary nipples [21, 23].

We propose that maintenance and/or enhancement of Wnt pathway activity is the critical molecular mechanism whereby Eda induces the formation of mammary placodes. Our conclusion is based on the following findings: 1) Canonical Wnt signaling is absolutely necessary for mammary placode induction and genetic deletion of Wnt pathway antagonists (Lrp4, Sostdc1) causes more epidermal cells to adopt mammary cell fate along the mammary line [3, 31, 49]; 2) Several Wnt pathway genes show an early upregulation at the prospective site of ectopic mammary placodes in *K14-Eda* embryos; and 3) Pharmacological inhibition of Wnt signaling suppresses formation of supernumerary placodes in *K14-Eda* mammary explants in a dose-dependent manner at doses that however, do not yet prevent formation of endogenous placodes. Eda and Wnt signaling pathways are intertwined during development of several ectodermal organs [24, 34, 52, 55, 56]. In primary hair placodes, Wnt/β-cat signaling enhances *Edar* expression which in turn is required for upregulation of *Wnt10a/b* to levels high enough for placode morphogenesis to proceed. In the absence of Eda, placode formation is halted at a rudimentary ‘pre-placode’ stage characterized by severely reduced levels of Wnt activation [19, 34, 52, 57]. However, mammary placodes are largely insensitive to loss of Eda. Interestingly, *Lgr4* deficient embryos display a similar defect in primary hair placodes as Eda null embryos [58] but all mammary glands form in *Lgr4* deficient mice [59]. Collectively, these data indicate that in the absence of Eda, cues other than Eda/NF-κB are responsible for maintenance of *Wnt10a/b/Lgr4* expression and thereby sufficient Wnt signaling activity to support early mammary morphogenesis. Alternatively, other Wnt ligands/Lgrs that are insensitive to Eda levels may have a more critical role in mammary primordia than in hair placodes.

### Regulation of mammary gland position and number–a role for Eda?

The number of mammary glands is usually considered to be a species-typical invariant trait [60–62] indicating that the balance between mammary fate promoting signals must be tightly balanced with inhibitory cues to ensure the development of the correct number of mammary glands. Yet, in some species such as the pig, dairy cattle, and multimammate mice (a.k.a. African soft-furred rat), *Mastomys natalensis* and its closely related species, a notable intraspecific variability has been observed [60, 63, 64]. Even in humans, accessory nipples and breast tissue are found at relatively high prevalence (estimates ranging from 0.2% to 5.6%) [65, 66]. These findings show that the mammary line has the capacity to produce more than the species-typical number of organs. Misregulation of the Eda/Wnt pathway could offer an explanation for some sporadic cases of polythelia or absence of breast.

The number and location of mammary glands vary widely between mammals [60, 62, 67]. In humans and other primates mammary glands are located at the thoracic region, in most ungulates at the inguinal region, in mice, cats and dogs at both regions, whereas pigs have them along the entire length of the milk line. Usually the number of pairs corresponds to the average number of offspring born at a time [61, 67]. Highest mammary gland numbers are seen in some marsupials and domesticated pigs, whereas mice and rats have maximally 6 pairs [60–62]. Multimammate mice are a striking exception with 8 to 12 pairs, or even more, scattered throughout the mammary line [64, 68] thereby greatly resembling *K14-Eda* mice. However, it is not known whether the milk line is expanded anteriorly as in *K14-Eda* mice. Changes in the Eda pathway activity have been linked to intraspecies evolutionary adaptations in the numbers of skin appendages in two species: the amount of armor plates in marine vs. freshwater threespine sticklebacks and the sweat gland density in modern human populations [69, 70]. It is tempting to speculate that differential expression levels of the Eda pathway components account for some of the interspecific differences observed in the number and position of mammary glands.

## Materials and Methods

### Animals

The generation and genotyping of the following mouse strains have been described: *K14-Eda* [26], *IκBαΔN* [19], *Eda* null (*Tabby*) (Jackson Laboratories; stock no. 000314), TOP-gal (Jackson laboratories; stock no. 004623), K17-GFP, *Foxi3*-deficient, and NF-κB reporter mice [24, 47, 71, 72]. *K14-Eda*, Foxi3-deficient, K17-GFP, and NF-κB rep mice were maintained on the C57Bl/6 background. *IκBαΔN* mice were bred in the C57BL/6 or a mixed C57BL/6 and FVB background. *Eda* null and TOP-Gal mice were on B6CBA and NMRI backgrounds, respectively. The appearance of a vaginal plug was considered the embryonic day (E) 0.5. The age of the embryos were further staged according to the limb morphogenesis [73] and other external criteria.

### Ethics statement

All mouse experiments were approved by the local ethics committee and National Animal Experiment Board of Finland under licenses KEK13-020 and ESAVI/2984-04.10.07–2014. The mice were sacrificed with CO_2_ followed by cervical dislocation.

### Histology, X-gal and carmine alum staining

Embryos or dissected tissues were fixed overnight in 4% PFA at 4°C, processed through rising ethanol series and xylene into paraffin and sectioned at 5 μm. Whole mount X-gal staining was done according to a published protocol [74]. The samples were postfixed with 4% PFA. When sectioned, the counterstain was performed with Nuclear fast red. Processing of the mammary glands and the Carmine alum staining was performed as previously described [24]. Whole embryos and tissues were photographed using the Olympus SZX9 stereomicroscope and slides with the Zeiss Imager.M2.

### Immunohistochemistry

For the keratin2e immunostaining, sections were deparaffinised and citrate-treated in 6mM sodium-citrate buffer (pH 6). The blocking was done with 5% goat serum in 3% BSA in PBS. The samples were incubated overnight with a primary mouse antibody against keratin2e (10R-C166a, 1:200, Fitzgerald) followed by a goat anti-mouse-HRP secondary antibody (1:500; Jackson Immuno Research). Detection was done with the Vectastain Elite ABC Kit (Vector Laboratories) and counterstain with haematoxylin. The whole mount immunostaining for Madcam1 was performed with a primary rat antibody against Madcam1 (550556, 1:25; BD Pharmingen) and a secondary anti-rat-HRP antibody (1:200, Santa Cruz Biotechnology). The DAB substrate kit for peroxidase (Vector Laboratories) was used for detection. Unspecific staining was blocked with 1% dry milk in 1xPBS/0.1% Tween-20.

### Organ culture

The ventrolateral skins that contained the mammary forming region and at least the endogenous buds 2, 3 and 4 were dissected from E12.5 embryos and half embryo explants were prepared from E11.0 embryos as indicated in the text. The explants were cultured for 1 to 2 days in a Trowell-type culture setting [24, 52]. The medium consisted of 1:1 mixture of DMEM and F12 (Ham’s Nutrient Mix: Life Technologies) and was supplemented with 10% (vol/vol) FCS (PAA Laboratories), 2 mM l-glutamine, penicillin-streptomycin and ascorbic acid (75 mg/L). When indicated, recombinant Eda protein (Fc-Eda-A1) [75] was added to the growth medium to achieve a final concentration of 250ng/mL. Wnt inhibitor XAV939 in DMSO (Stemgent) was used as 10 μM or 40μM concentrations. Two separate stock solutions were generated for the inhibitor in order to avoid DMSO concentrations higher than 0.25% in the culture medium. Each time, one side of the embryo was used as the control and the other was treated with XAV939.

### In situ hybridization

The embryos or tissue culture samples were fixed overnight in 4% PFA at 4°C and processed for whole mount in situ hybridization or for paraffin-embedding. The whole mount in situ hybridization was performed with inSituPro robot (Intavis AG). The following digoxigenin-labelled RNA probes were used: *PTHrP* [76], *Wnt10b* [77], *Wnt10a* [78], *Lef1*, *β-catenin*, *Shh* [79], *Tbx3*, *Nrg3* [15], *Kremen2* (nucleotides 1306–1703 of NM_028416.2), *Mmp9* (nucleotides 527–1131 of NM_013599.3) and *Lgr4* (nucleotides 3408–3823 of NM_172671.2). The detection was achieved by using BM Purple AP substrate Precipitating Solution (Boehringer Mannheim). Radioactive in situ hybridization on paraffin sections was carried out according to previously described protocols using ^35^S-UTP labelled (Amersham) probe specific to *Edar* [80].

### Hanging drop experiment and quantitative RT-PCR

The hanging drop culture has been described in detail elsewhere [24, 52]. In short, two pools of 15–20 E13.5 *Eda-/-* mammary buds from 4 or 5 embryos were collected for each sample pair: one pool was treated with 250 ng/mL of Fc-Eda for 4h, whereas the other one was maintained in a control medium for 4h. RNA extraction and cDNA synthesis was performed as described previously [24, 52]. qRT-PCR was done in a LightCycler 480 (Roche, Indianapolis, IA) and the following analysis was done with software provided by the manufacturer. The expression data were normalized against *Ranbp1* gene. For primer sequences see (S2 Table).

### Statistical analyses

Unpaired Student’s t- test was used for statistical analysis of all data. P-values of ≤0.05 were considered to be statistically significant.

### Microarray

E13.5 mammary buds were dissected from *Eda-/-* embryos and used either as a control or exposed to 250 ng/mL of Fc-Eda as described above. 15–20 mammary buds were pooled in one sample, and three biological replicates were collected. RNA was extracted as previously described [24, 28] and RNA quality was monitored using a 2100 Bioanalyzer (Agilent Technologies). RNAs were processed and hybridized on Affymetrix Mouse Exon 1.0 ST arrays (Santa Clara, CA) in the Biomedicum Functional Genomics unit (University of Helsinki, Finland). Significance analysis between treated and control samples was done using three statistical tests. In each test, a paired t-test (pairing was done over treatment-control pairs) was applied to the data. Differentially expressed genes were detected using Limma, IBMT (intensity based moderated t-test), and Cyber-T. All methods were applied with default parameters. Obtained p-values were adjusted for multiple testing using Storey’s q-value method. The data discussed in this publication have been deposited in NCBI's Gene Expression Omnibus [81] and are accessible through GEO Series accession number GSE69781 (http://www.ncbi.nlm.nih.gov/geo/query/acc.cgi?acc=GSE69781)

## Supporting Information

S1 Fig NF-κB reporter expression localizes gradually to the emerging mammary placodes. Whole-mount X-gal stained NF-κB reporter embryos at E11.0 (A), E11.25(-) (B), E11.25 (C), and E11.5 (D) reveal NF-κB activity in mammary placodes and the mammary forming region. Reporter expression was first observed in placodes 3 (mp3) and 1 (mp1) (A) and it gradually became more focal as the placodes formed (B-D). Note that a stripe of low-level reporter positive cells links all the mammary placodes at E11.25 and E11.5. (Scale bar: 500 μm)
(TIF)

S2 Fig Expression of Madcam1 and *Mmp9* correlates with Eda. Whole mount analysis of Madcam1 protein (A) and *Mmp9* mRNA expression (B, B’) in *Eda-/-*, WT and *K14-Eda* embryos at E13.5. Note prominent expression of *Mmp9* also in developing vibrissae (arrows) in *K14-Eda* embryos (B'). (Scale bar: 500 μm.).
(TIF)

S3 Fig Eda-induced supernumerary mammary placodes form in Foxi3-deficient background. (A-D) Mammary buds were visualized by whole mount in situ hybridization with a *Wnt10b* specific probe in wt (A), *Foxi3*-/- (B), *K14-Eda* (C), and *Foxi3*-/-;*K14-Eda* (D) embryos at E13.75. Arrows highlight the supernumerary mammary placodes in *K14-Eda* and in compound *Foxi3*-/-;*K14-Eda* embryos at E13.75. (Scale bar: 500 μm.).
(TIF)

S4 Fig Ex vivo application of Fc-Eda protein induces formation of supernumerary mammary buds that express typical mammary primordia markers, but not a hair placode-specific marker. (A-D) In situ hybridization for *Wnt10b* (A), *Krm2* (B), *PTHrP* (C), and *Shh* (D) of E12.5 wild-type explants cultured in the control medium or exposed to 250 ng/mL of Fc-Eda for 2 days. (Scale bar: 100 μm).
(TIF)

S5 Fig High Wnt inhibitor concentration does not block endogenous mammary placode development. E11.0 K17-GFP half embryo explants were cultured for 48 hours to visualize mammary placode development in the presence of Wnt inhibitor XAV939. In control and treated specimen, 7/8 explants had clearly recognizable mammary buds after 2 days of culture. 40 μM concentration of the inhibitor reduced the size of endogenous mammary rudiments. (A) Control and XAV939 treated explants of the same embryo at the beginning of the culture period (E11.0+0d) and (B) same explants at the end of the culture period (E11.0+2d).
(TIF)

S1 Table The complete list of differentially expressed genes in E13.5 Eda-/- mammary buds after 4 hours treatment with the Fc-Eda protein. Only genes displaying a q-value <0.05 at least in one of the three statistical analyses are included. Log2 indicates (log2(Fc-Eda-treatment)-log2(control)).
(XLSX)

S2 Table Sequences of the qRT-PCR primers used in this study. (XLSX)

## References

[1] Propper AY, Howard BA, Veltmaat JM. (2013) Prenatal morphogenesis of mammary glands in mouse and rabbit. J Mammary Gland Biol Neoplasia 18(2): 93–104. 10.1007/s10911-013-9298-0 23736987 PMC3691486

[2] Veltmaat JM, Van Veelen W, Thiery JP, Bellusci S. (2004) Identification of the mammary line in mouse by Wnt10b expression. Dev Dyn 229(2): 349–356. 10.1002/dvdy.10441 14745960

[3] Chu EY, Hens J, Andl T, Kairo A, Yamaguchi TP, et al (2004) Canonical WNT signaling promotes mammary placode development and is essential for initiation of mammary gland morphogenesis. Development 131(19): 4819–4829. 10.1242/dev.01347 15342465

[4] Mailleux AA, Spencer-Dene B, Dillon C, Ndiaye D, Savona-Baron C, et al (2002) Role of FGF10/FGFR2b signaling during mammary gland development in the mouse embryo. Development 129(1): 53–60. 11782400 10.1242/dev.129.1.53

[5] Balinsky BI. (1950) On the prenatal growth of the mammary gland rudiment in the mouse. J Anat 84(3): 227–235. 15436328 PMC1273299

[6] Lee MY, Racine V, Jagadpramana P, Sun L, Yu W, et al (2011) Ectodermal influx and cell hypertrophy provide early growth for all murine mammary rudiments, and are differentially regulated among them by Gli3. PLoS One 6(10): e26242 10.1371/journal.pone.0026242 22046263 PMC3203106

[7] Biggs LC, Mikkola ML. (2014) Early inductive events in ectodermal appendage morphogenesis. Semin Cell Dev Biol 25–26: 11–21. 10.1016/j.semcdb.2014.01.007 24487243

[8] Pispa J, Thesleff I. (2003) Mechanisms of ectodermal organogenesis. Dev Biol 262(2): 195–205. 14550785 10.1016/s0012-1606(03)00325-7

[9] Cowin P, Wysolmerski J. (2010) Molecular mechanisms guiding embryonic mammary gland development. Cold Spring Harb Perspect Biol. 10.1101/cshperspect.a003251 PMC2869520 20484386

[10] Veltmaat JM, Relaix F, Le LT, Kratochwil K, Sala FG, et al (2006) Gli3-mediated somitic Fgf10 expression gradients are required for the induction and patterning of mammary epithelium along the embryonic axes. Development 133(12): 2325–2335. 133/12/2325 [pii]. 16720875 10.1242/dev.02394

[11] Hatsell SJ, Cowin P. (2006) Gli3-mediated repression of hedgehog targets is required for normal mammary development. Development 133(18): 3661–3670. dev.02542 [pii]. 16914490 10.1242/dev.02542

[12] Davenport TG, Jerome-Majewska LA, Papaioannou VE. (2003) Mammary gland, limb and yolk sac defects in mice lacking Tbx3, the gene mutated in human ulnar mammary syndrome. Development 130(10): 2263–2273. 12668638 10.1242/dev.00431

[13] Eblaghie MC, Song SJ, Kim JY, Akita K, Tickle C, et al (2004) Interactions between FGF and wnt signals and Tbx3 gene expression in mammary gland initiation in mouse embryos. J Anat 205(1): 1–13. 10.1111/j.0021-8782.2004.00309.x 15255957 PMC1571327

[14] Cho KW, Kim JY, Song SJ, Farrell E, Eblaghie MC, et al (2006) Molecular interactions between Tbx3 and Bmp4 and a model for dorsoventral positioning of mammary gland development. Proc Natl Acad Sci U S A 103(45): 16788–16793. 0604645103 [pii]. 17071745 10.1073/pnas.0604645103 PMC1636533

[15] Howard B, Panchal H, McCarthy A, Ashworth A. (2005) Identification of the scaramanga gene implicates Neuregulin3 in mammary gland specification. Genes Dev 19(17): 2078–2090. 19/17/2078 [pii]. 16140987 10.1101/gad.338505 PMC1199577

[16] Panchal H, Wansbury O, Parry S, Ashworth A, Howard B. (2007) Neuregulin3 alters cell fate in the epidermis and mammary gland. BMC Dev Biol 7: 105 1471-213X-7-105 [pii]. 17880691 10.1186/1471-213X-7-105 PMC2110892

[17] Mikkola ML. (2008) TNF superfamily in skin appendage development. Cytokine Growth Factor Rev 19(3–4): 219–230. 10.1016/j.cytogfr.2008.04.008 18495521

[18] Kowalczyk-Quintas C, Schneider P. (2014) Ectodysplasin A (EDA)—EDA receptor signalling and its pharmacological modulation. Cytokine Growth Factor Rev 25(2): 195–203. 10.1016/j.cytogfr.2014.01.004 24508088

[19] Schmidt-Ullrich R, Aebischer T, Hulsken J, Birchmeier W, Klemm U, et al (2001) Requirement of NF-kappaB/rel for the development of hair follicles and other epidermal appendices. Development 128(19): 3843–3853. 11585809 10.1242/dev.128.19.3843

[20] Haara O, Fujimori S, Schmidt-Ullrich R, Hartmann C, Thesleff I, et al (2011) Ectodysplasin and wnt pathways are required for salivary gland branching morphogenesis. Development 138(13): 2681–2691. 10.1242/dev.057711 21652647

[21] Clarke A, Phillips DI, Brown R, Harper PS. (1987) Clinical aspects of X-linked hypohidrotic ectodermal dysplasia. Arch Dis Child 62(10): 989–996. 2445301 10.1136/adc.62.10.989 PMC1778691

[22] Haghighi A, Nikuei P, Haghighi-Kakhki H, Saleh-Gohari N, Baghestani S, et al (2013) Whole-exome sequencing identifies a novel missense mutation in EDAR causing autosomal recessive hypohidrotic ectodermal dysplasia with bilateral amastia and palmoplantar hyperkeratosis. Br J Dermatol 168(6): 1353–1356. 10.1111/bjd.12151 23210707

[23] Megarbane H, Cluzeau C, Bodemer C, Fraitag S, Chababi-Atallah M, et al (2008) Unusual presentation of a severe autosomal recessive anhydrotic ectodermal dysplasia with a novel mutation in the EDAR gene. Am J Med Genet A 146A(20): 2657–2662. 10.1002/ajmg.a.32509 18816645

[24] Voutilainen M, Lindfors PH, Lefebvre S, Ahtiainen L, Fliniaux I, et al (2012) Ectodysplasin regulates hormone-independent mammary ductal morphogenesis via NF-kappaB. Proc Natl Acad Sci U S A 109(15): 5744–5749. 10.1073/pnas.1110627109 22451941 PMC3326516

[25] Pispa J, Pummila M, Barker PA, Thesleff I, Mikkola ML. (2008) Edar and troy signalling pathways act redundantly to regulate initiation of hair follicle development. Hum Mol Genet 17(21): 3380–3391. 10.1093/hmg/ddn232 18689798

[26] Mustonen T, Pispa J, Mikkola ML, Pummila M, Kangas AT, et al (2003) Stimulation of ectodermal organ development by ectodysplasin-A1. Dev Biol 259(1): 123–136. 12812793 10.1016/s0012-1606(03)00157-x

[27] Mustonen T, Ilmonen M, Pummila M, Kangas AT, Laurikkala J, et al (2004) Ectodysplasin A1 promotes placodal cell fate during early morphogenesis of ectodermal appendages. Development 131(20): 4907–4919. 10.1242/dev.01377 15371307

[28] Fliniaux I, Mikkola ML, Lefebvre S, Thesleff I. (2008) Identification of dkk4 as a target of eda-A1/edar pathway reveals an unexpected role of ectodysplasin as inhibitor of wnt signalling in ectodermal placodes. Dev Biol 320(1): 60–71. 10.1016/j.ydbio.2008.04.023 18508042

[29] Mahler B, Gocken T, Brojan M, Childress S, Spandau DF, et al (2004) Keratin 2e: A marker for murine nipple epidermis. Cells Tissues Organs 176(4): 169–177. 10.1159/000077033 15118396

[30] Baud V, Karin M. (2009) Is NF-kappaB a good target for cancer therapy? hopes and pitfalls. Nat Rev Drug Discov 8(1): 33–40. 10.1038/nrd2781 19116625 PMC2729321

[31] Narhi K, Tummers M, Ahtiainen L, Itoh N, Thesleff I, et al (2012) Sostdc1 defines the size and number of skin appendage placodes. Dev Biol 364(2): 149–161. 22509524 10.1016/j.ydbio.2012.01.026

[32] Boras-Granic K, Hamel PA. (2013) Wnt-signalling in the embryonic mammary gland. J Mammary Gland Biol Neoplasia 18(2): 155–163. 10.1007/s10911-013-9280-x 23660702

[33] Jerome-Majewska LA, Jenkins GP, Ernstoff E, Zindy F, Sherr CJ, et al (2005) Tbx3, the ulnar-mammary syndrome gene, and Tbx2 interact in mammary gland development through a p19Arf/p53-independent pathway. Dev Dyn 234(4): 922–933. 10.1002/dvdy.20575 16222716

[34] Zhang Y, Tomann P, Andl T, Gallant NM, Huelsken J, et al (2009) Reciprocal requirements for EDA/EDAR/NF-kappaB and wnt/beta-catenin signaling pathways in hair follicle induction. Dev Cell 17(1): 49–61. 10.1016/j.devcel.2009.05.011 19619491 PMC2859042

[35] Lefebvre S, Fliniaux I, Schneider P, Mikkola ML. (2012) Identification of ectodysplasin target genes reveals the involvement of chemokines in hair development. J Invest Dermatol 132(4): 1094–1102. 10.1038/jid.2011.453 22277947

[36] Wiseman BS, Sternlicht MD, Lund LR, Alexander CM, Mott J, et al (2003) Site-specific inductive and inhibitory activities of MMP-2 and MMP-3 orchestrate mammary gland branching morphogenesis. J Cell Biol 162(6): 1123–1133. 10.1083/jcb.200302090 12975354 PMC2172848

[37] Drogemuller C, Karlsson EK, Hytonen MK, Perloski M, Dolf G, et al (2008) A mutation in hairless dogs implicates FOXI3 in ectodermal development. Science 321(5895): 1462 10.1126/science.1162525 18787161

[38] Shirokova V, Jussila M, Hytonen MK, Perala N, Drogemuller C, et al (2013) Expression of Foxi3 is regulated by ectodysplasin in skin appendage placodes. Dev Dyn 242(6): 593–603. 10.1002/dvdy.23952 23441037

[39] Mao B, Niehrs C. (2003) Kremen2 modulates Dickkopf2 activity during wnt/LRP6 signaling. Gene 302(1–2): 179–183. 12527209 10.1016/s0378-1119(02)01106-x

[40] de Lau WB, Snel B, Clevers HC. (2012) The R-spondin protein family. Genome Biol 13(3): 242-2012-13-3-242. 10.1186/gb-2012-13-3-242 22439850 PMC3439965

[41] Carmon KS, Gong X, Lin Q, Thomas A, Liu Q. (2011) R-spondins function as ligands of the orphan receptors LGR4 and LGR5 to regulate wnt/beta-catenin signaling. Proc Natl Acad Sci U S A 108(28): 11452–11457. 10.1073/pnas.1106083108 21693646 PMC3136304

[42] Weng J, Luo J, Cheng X, Jin C, Zhou X, et al (2008) Deletion of G protein-coupled receptor 48 leads to ocular anterior segment dysgenesis (ASD) through down-regulation of Pitx2. Proc Natl Acad Sci U S A 105(16): 6081–6086. 10.1073/pnas.0708257105 18424556 PMC2329706

[43] Zhang X, Ibrahimi OA, Olsen SK, Umemori H, Mohammadi M, et al (2006) Receptor specificity of the fibroblast growth factor family. the complete mammalian FGF family. J Biol Chem 281(23): 15694–15700. M601252200 [pii]. 16597617 10.1074/jbc.M601252200 PMC2080618

[44] Voutilainen M, Lindfors PH, Mikkola ML. (2013) Protocol: Ex vivo culture of mouse embryonic mammary buds. J Mammary Gland Biol Neoplasia 18(2): 239–245. 10.1007/s10911-013-9288-2 23674216

[45] Michno K, Boras-Granic K, Mill P, Hui CC, Hamel PA. (2003) Shh expression is required for embryonic hair follicle but not mammary gland development. Dev Biol 264(1): 153–165. S0012160603004019 [pii]. 14623238 10.1016/s0012-1606(03)00401-9

[46] Huang SM, Mishina YM, Liu S, Cheung A, Stegmeier F, et al (2009) Tankyrase inhibition stabilizes axin and antagonizes wnt signalling. Nature 461(7264): 614–620. 10.1038/nature08356 19759537

[47] Bianchi N, Depianto D, McGowan K, Gu C, Coulombe PA. (2005) Exploiting the keratin 17 gene promoter to visualize live cells in epithelial appendages of mice. Mol Cell Biol 25(16): 7249–7259. 25/16/7249 [pii]. 16055733 10.1128/MCB.25.16.7249-7259.2005 PMC1190235

[48] Rohrschneider LR, Custodio JM, Anderson TA, Miller CP, Gu H. (2005) The intron 5/6 promoter region of the ship1 gene regulates expression in stem/progenitor cells of the mouse embryo. Dev Biol 283(2): 503–521. S0012-1606(05)00270-8 [pii]. 15978570 10.1016/j.ydbio.2005.04.032

[49] Ahn Y, Sims C, Logue JM, Weatherbee SD, Krumlauf R. (2013) Lrp4 and wise interplay controls the formation and patterning of mammary and other skin appendage placodes by modulating wnt signaling. Development 140(3): 583–593. 10.1242/dev.085118 23293290 PMC6514302

[50] Hehlgans T, Pfeffer K. (2005) The intriguing biology of the tumour necrosis factor/tumour necrosis factor receptor superfamily: Players, rules and the games. Immunology 115(1): 1–20. IMM2143 [pii]. 15819693 10.1111/j.1365-2567.2005.02143.x PMC1782125

[51] Kumar A, Eby MT, Sinha S, Jasmin A, Chaudhary PM. (2001) The ectodermal dysplasia receptor activates the nuclear factor-kappaB, JNK, and cell death pathways and binds to ectodysplasin A. J Biol Chem 276(4): 2668–2677. 10.1074/jbc.M008356200 11035039

[52] Fliniaux I, Mikkola ML, Lefebvre S, Thesleff I. (2008) Identification of dkk4 as a target of eda-A1/edar pathway reveals an unexpected role of ectodysplasin as inhibitor of wnt signalling in ectodermal placodes. Dev Biol 320(1): 60–71. 10.1016/j.ydbio.2008.04.023 18508042

[53] Kondo S, Miura T. (2010) Reaction-diffusion model as a framework for understanding biological pattern formation. Science 329(5999): 1616–1620. 10.1126/science.1179047 20929839

[54] Painter KJ, Hunt GS, Wells KL, Johansson JA, Headon DJ. (2012) Towards an integrated experimental-theoretical approach for assessing the mechanistic basis of hair and feather morphogenesis. Interface Focus 2(4): 433–450. 10.1098/rsfs.2011.0122 23919127 PMC3363042

[55] Arte S, Parmanen S, Pirinen S, Alaluusua S, Nieminen P. (2013) Candidate gene analysis of tooth agenesis identifies novel mutations in six genes and suggests significant role for WNT and EDA signaling and allele combinations. PLoS One 8(8): e73705 10.1371/journal.pone.0073705 23991204 PMC3750013

[56] Cui CY, Yin M, Sima J, Childress V, Michel M, et al (2014) Involvement of wnt, eda and shh at defined stages of sweat gland development. Development 141(19): 3752–3760. 10.1242/dev.109231 25249463 PMC4197578

[57] Schmidt-Ullrich R, Tobin DJ, Lenhard D, Schneider P, Paus R, et al (2006) NF-kappaB transmits eda A1/EdaR signalling to activate shh and cyclin D1 expression, and controls post-initiation hair placode down growth. Development 133(6): 1045–1057. dev.02278 [pii]. 16481354 10.1242/dev.02278

[58] Mohri Y, Kato S, Umezawa A, Okuyama R, Nishimori K. (2008) Impaired hair placode formation with reduced expression of hair follicle-related genes in mice lacking Lgr4. Dev Dyn 237(8): 2235–2242. 10.1002/dvdy.21639 18651655

[59] Wang Y, Dong J, Li D, Lai L, Siwko S, et al (2013) Lgr4 regulates mammary gland development and stem cell activity through the pluripotency transcription factor Sox2. Stem Cells 31(9): 1921–1931. 10.1002/stem.1438 23712846 PMC3934111

[60] Bresslau E. (1920) The mammary apparatus of the mammalia: In the light of ontogenesis and phylogenesis London: Methuen & Co.

[61] Gilbert AN. (1986) Mammary number and litter size in rodentia: The "one-half rule". Proc Natl Acad Sci U S A 83(13): 4828–4830. 16593720 10.1073/pnas.83.13.4828 PMC323835

[62] Veltmaat JM, Ramsdell AF, Sterneck E. (2013) Positional variations in mammary gland development and cancer. J Mammary Gland Biol Neoplasia 18(2): 179–188. 10.1007/s10911-013-9287-3 23666389 PMC3691492

[63] Gifford W. (1934) The occurrence of polythelia in dairy cattle. Journal of Dairy Science 17(8): 559–569.

[64] Lecompte E, Granjon L, Denys C. (2002) The phylogeny of the *Praomys* complex (rodentia: Muridae) and its phylogeographic implications. Journal of Zoological Systematics and Evolutionary Research 40(1): 8–25.

[65] Kajava Y. (1915) The proportions of supernumerary nipples in the finnish population. Duodecim 1: 143–70.

[66] Loukas M, Clarke P, Tubbs RS. (2007) Accessory breasts: A historical and current perspective. Am Surg 73(5): 525–528. 17521013

[67] Veltmaat JM, Mailleux AA, Thiery JP, Bellusci S. (2003) Mouse embryonic mammogenesis as a model for the molecular regulation of pattern formation. Differentiation 71(1): 1–17. 10.1046/j.1432-0436.2003.700601.x 12558599

[68] Brambell F, Davis D, Jarvis J. (1941) Reproduction of the multimammate mouse (*Mastomys erythroleucus* temm.) of sierra leone. Proceedings of the Zoological Society of London B 111(1–2): 1–11.

[69] Colosimo PF, Hosemann KE, Balabhadra S, Villarreal G Jr, Dickson M, et al (2005) Widespread parallel evolution in sticklebacks by repeated fixation of ectodysplasin alleles. Science 307(5717): 1928–1933. 307/5717/1928 [pii]. 15790847 10.1126/science.1107239

[70] Kamberov YG, Wang S, Tan J, Gerbault P, Wark A, et al (2013) Modeling recent human evolution in mice by expression of a selected EDAR variant. Cell 152(4): 691–702. 10.1016/j.cell.2013.01.016 23415220 PMC3575602

[71] Bhakar AL, Tannis LL, Zeindler C, Russo MP, Jobin C, et al (2002) Constitutive nuclear factor-kappa B activity is required for central neuron survival. J Neurosci 22(19): 8466–8475. 12351721 10.1523/JNEUROSCI.22-19-08466.2002 PMC6757785

[72] Edlund RK, Ohyama T, Kantarci H, Riley BB, Groves AK. (2014) Foxi transcription factors promote pharyngeal arch development by regulating formation of FGF signaling centers. Dev Biol 390(1): 1–13. 10.1016/j.ydbio.2014.03.004 24650709 PMC4013273

[73] Martin P. (1990) Tissue patterning in the developing mouse limb. Int J Dev Biol 34(3): 323–336. 1702679

[74] Pispa J, Pummila M, Barker PA, Thesleff I, Mikkola ML. (2008) Edar and troy signalling pathways act redundantly to regulate initiation of hair follicle development. Hum Mol Genet 17(21): 3380–3391. 10.1093/hmg/ddn232 18689798

[75] Gaide O, Schneider P. (2003) Permanent correction of an inherited ectodermal dysplasia with recombinant EDA. Nat Med 9(5): 614–618. 10.1038/nm861 12692542

[76] Liu JG, Tabata MJ, Yamashita K, Matsumura T, Iwamoto M, et al (1998) Developmental role of PTHrP in murine molars. Eur J Oral Sci 106 Suppl 1: 143–146. 9541217 10.1111/j.1600-0722.1998.tb02167.x

[77] Wang J, Shackleford GM. (1996) Murine Wnt10a and Wnt10b: Cloning and expression in developing limbs, face and skin of embryos and in adults. Oncogene 13(7): 1537–1544. 8875992

[78] Dassule HR, McMahon AP. (1998) Analysis of epithelial-mesenchymal interactions in the initial morphogenesis of the mammalian tooth. Dev Biol 202(2): 215–227. S0012-1606(98)98992-8 [pii]. 9769173 10.1006/dbio.1998.8992

[79] Laurikkala J, Pispa J, Jung HS, Nieminen P, Mikkola M, et al (2002) Regulation of hair follicle development by the TNF signal ectodysplasin and its receptor edar. Development 129(10): 2541–2553. 11973284 10.1242/dev.129.10.2541

[80] Laurikkala J, Mikkola M, Mustonen T, Aberg T, Koppinen P, et al (2001) TNF signaling via the ligand-receptor pair ectodysplasin and edar controls the function of epithelial signaling centers and is regulated by wnt and activin during tooth organogenesis. Dev Biol 229(2): 443–455. 10.1006/dbio.2000.9955 11203701

[81] Edgar R, Domrachev M, Lash AE. (2002) Gene expression omnibus: NCBI gene expression and hybridization array data repository. Nucleic Acids Res 30(1): 207–210. 11752295 10.1093/nar/30.1.207 PMC99122

