# Supplementary figures and images for: Ectodysplasin/NF-κB Promotes Mammary Cell Fate via Wnt/β-catenin Pathway
Source: PLoS Genet. 2015 Nov 18;11(11):e1005676. doi: 10.1371/journal.pgen.1005676 (PMC4651331; doi:10.1371/journal.pgen.1005676)

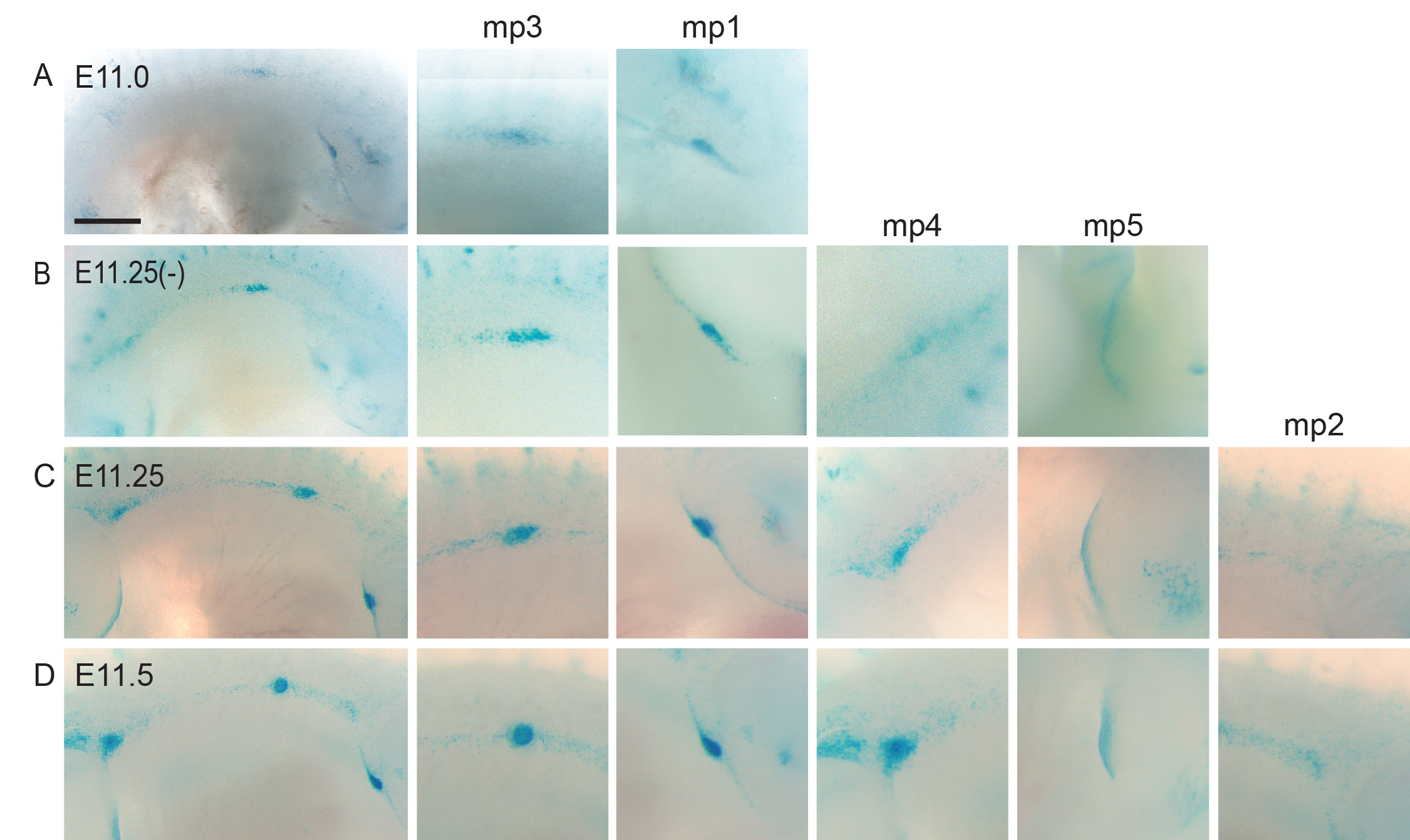

Supplement: S1 Fig — Whole-mount X-gal stained NF-κB reporter embryos at E11.0 (A), E11.25(-) (B), E11.25 (C), and E11.5 (D) reveal NF-κB activity in mammary placodes and the mammary forming region. Reporter expression was first observed in placodes 3 (mp3) and 1 (mp1) (A) and it gradually became more focal as the placodes formed (B-D). Note that a stripe of low-level reporter positive cells links all the mammary placodes at E11.25 and E11.5. (Scale bar: 500 μm) (TIF) [file pgen.1005676.s001.tif]

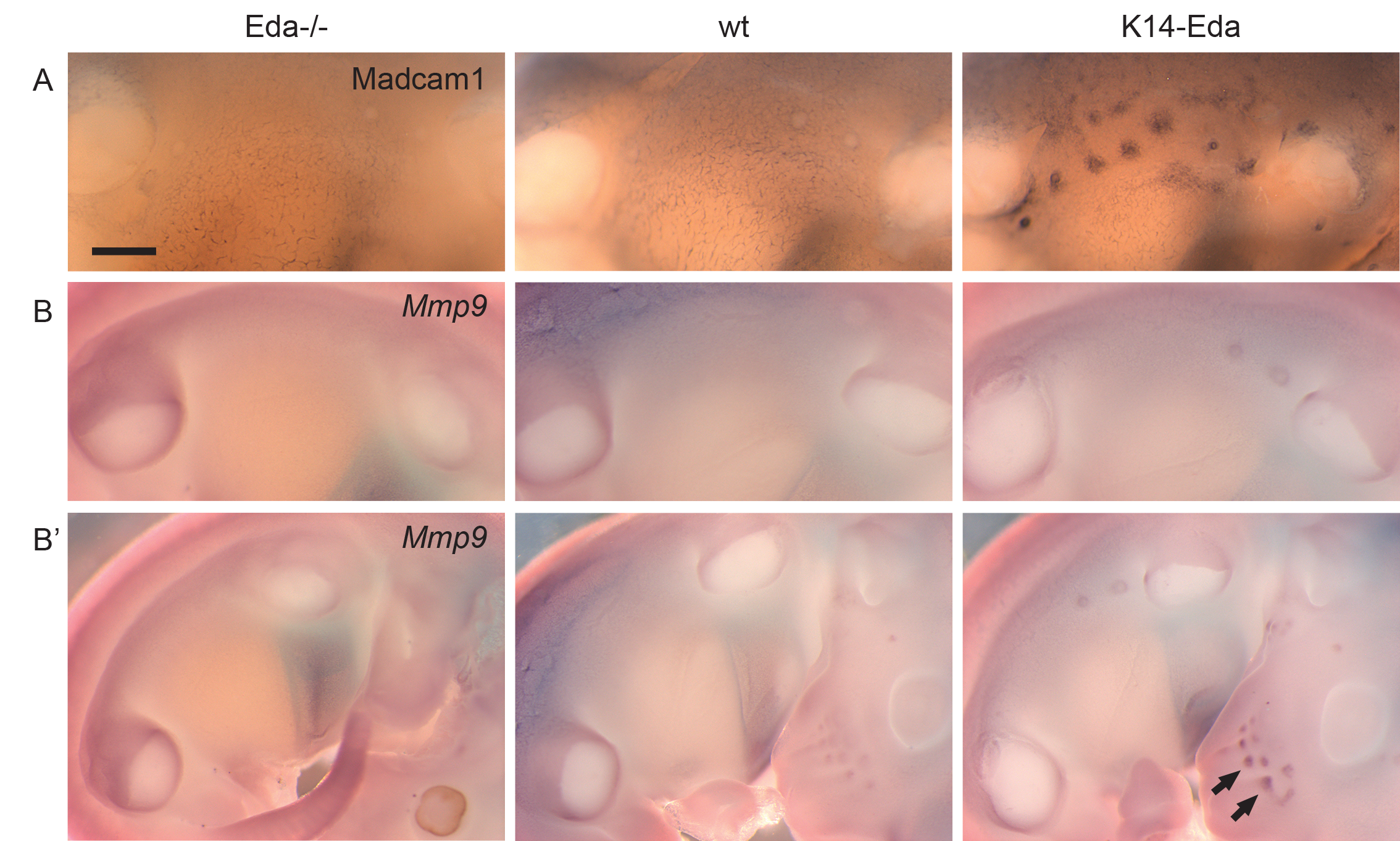

Supplement: S2 Fig — Whole mount analysis of Madcam1 protein (A) and Mmp9 mRNA expression (B, B’) in Eda-/-, WT and K14-Eda embryos at E13.5. Note prominent expression of Mmp9 also in developing vibrissae (arrows) in K14-Eda embryos (B'). (Scale bar: 500 μm.). (TIF) [file pgen.1005676.s002.tif]

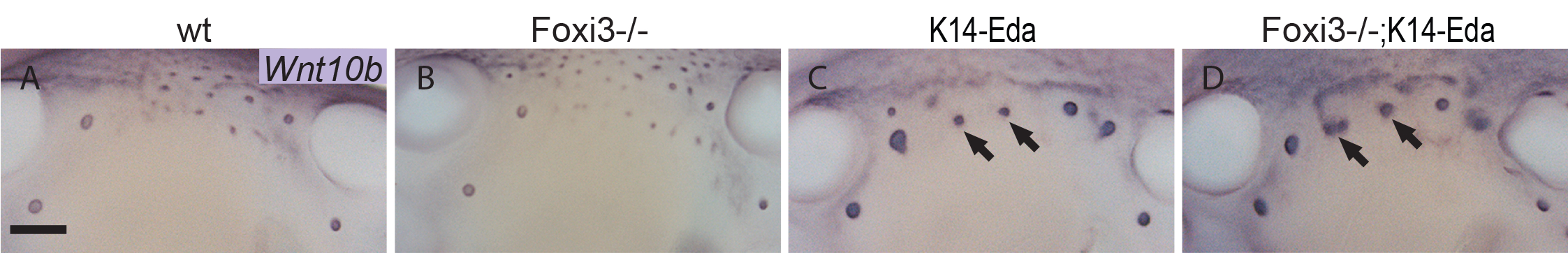

Supplement: S3 Fig — (A-D) Mammary buds were visualized by whole mount in situ hybridization with a Wnt10b specific probe in wt (A), Foxi3-/- (B), K14-Eda (C), and Foxi3-/-;K14-Eda (D) embryos at E13.75. Arrows highlight the supernumerary mammary placodes in K14-Eda and in compound Foxi3-/-;K14-Eda embryos at E13.75. (Scale bar: 500 μm.). (TIF) [file pgen.1005676.s003.tif]

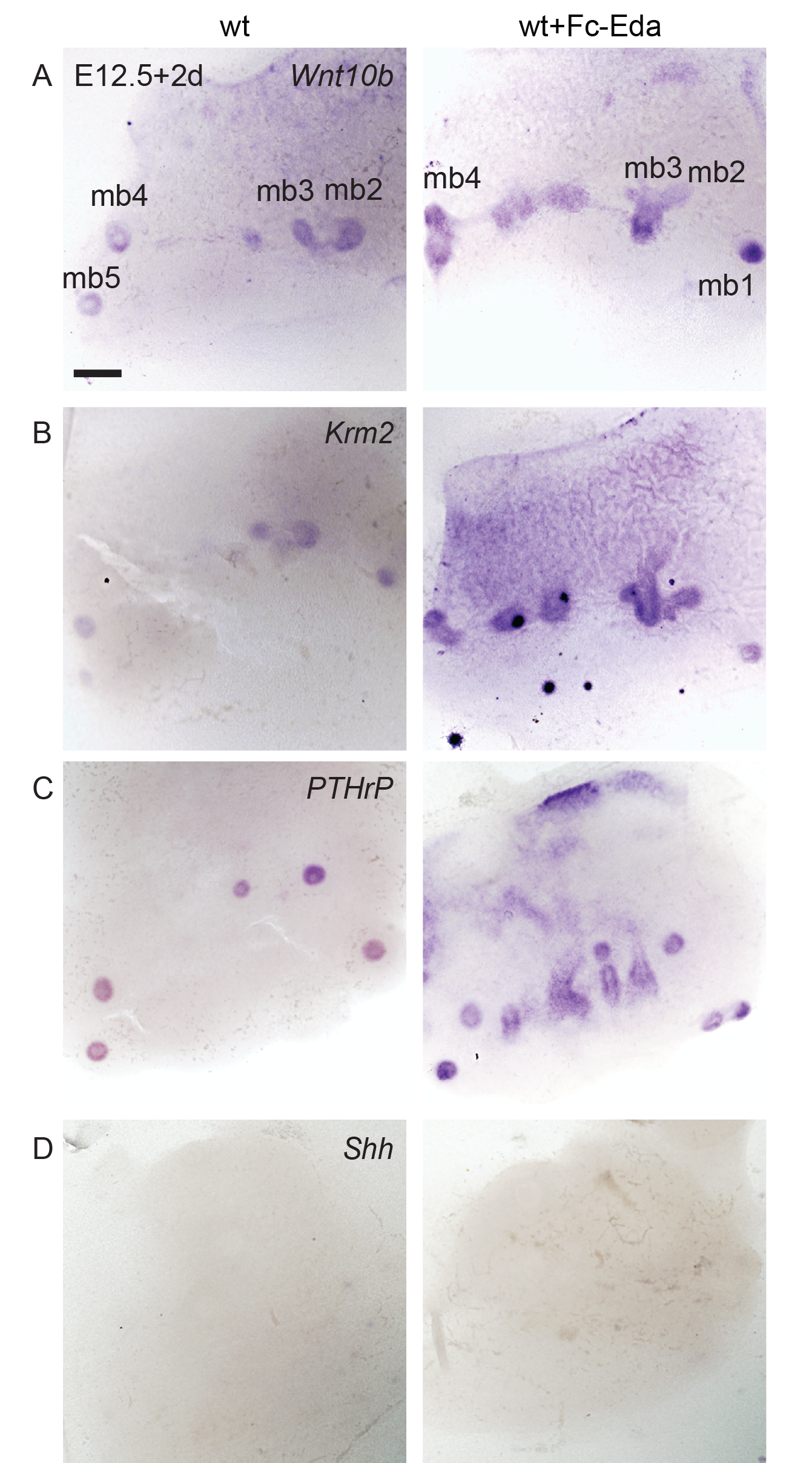

Supplement: S4 Fig — (A-D) In situ hybridization for Wnt10b (A), Krm2 (B), PTHrP (C), and Shh (D) of E12.5 wild-type explants cultured in the control medium or exposed to 250 ng/mL of Fc-Eda for 2 days. (Scale bar: 100 μm). (TIF) [file pgen.1005676.s004.tif]

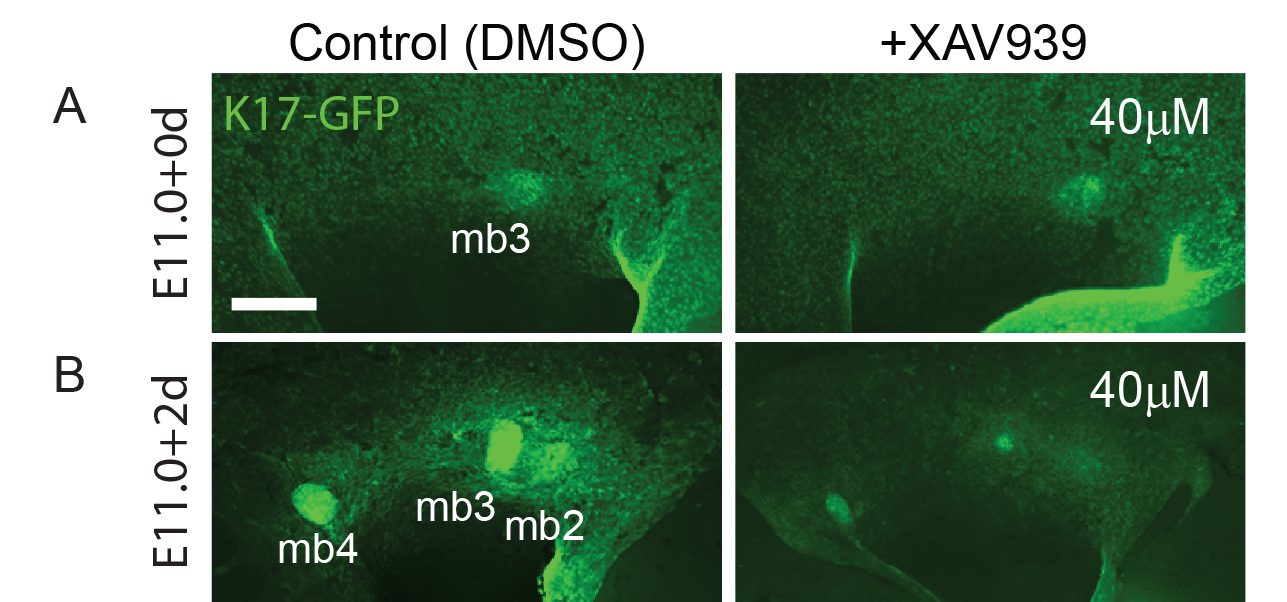

Supplement: S5 Fig — E11.0 K17-GFP half embryo explants were cultured for 48 hours to visualize mammary placode development in the presence of Wnt inhibitor XAV939. In control and treated specimen, 7/8 explants had clearly recognizable mammary buds after 2 days of culture. 40 μM concentration of the inhibitor reduced the size of endogenous mammary rudiments. (A) Control and XAV939 treated explants of the same embryo at the beginning of the culture period (E11.0+0d) and (B) same explants at the end of the culture period (E11.0+2d). (TIF) [file pgen.1005676.s005.tif]
